# *De novo* genome assembly of the red silk cotton tree (*Bombax ceiba*)

**DOI:** 10.1093/gigascience/giy051

**Published:** 2018-05-10

**Authors:** Yong Gao, Haibo Wang, Chao Liu, Honglong Chu, Dongqin Dai, Shengnan Song, Long Yu, Lihong Han, Yi Fu, Bin Tian, Lizhou Tang

**Affiliations:** 1 Center for Yunnan Plateau Biological Resources Protection and Utilization, College of Biological Resource and Food Engineering, Qujing Normal University, Qujing, Yunnan, 655011, China; 2 Key Laboratory of Biodiversity Conservation in Southwest China, State Forestry Administration, Southwest Forestry University, Kunming 650224, China; 3 Key Laboratory of Biodiversity and Biogeography, Kunming Institute of Botany, Chinese Academy of Sciences, Kunming 650204, China; 4 State Key Laboratory of Genetic Resources and Evolution, Kunming Institute of Zoology, Chinese Academy of Sciences, Kunming 650223, China; 5 Nextomics Biosciences Institute, Wuhan, Hubei 430000, China

**Keywords:** *Bombax ceiba*, genome assembly, annotation, evolution

## Abstract

**Background:**

*Bombax ceiba* L. (the red silk cotton tree) is a large deciduous tree that is distributed in tropical and sub-tropical Asia as well as northern Australia. It has great economic and ecological importance, with several applications in industry and traditional medicine in many Asian countries. To facilitate further utilization of this plant resource, we present here the draft genome sequence for *B. ceiba*.

**Findings:**

We assembled a relatively intact genome of *B. ceiba* by using PacBio single-molecule sequencing and BioNano optical mapping technologies. The final draft genome is approximately 895 Mb long, with contig and scaffold N50 sizes of 1.0 Mb and 2.06 Mb, respectively.

**Conclusions:**

The high-quality draft genome assembly of *B. ceiba* will be a valuable resource enabling further genetic improvement and more effective use of this tree species.

## Data Description

### Introduction

**Figure 1:**
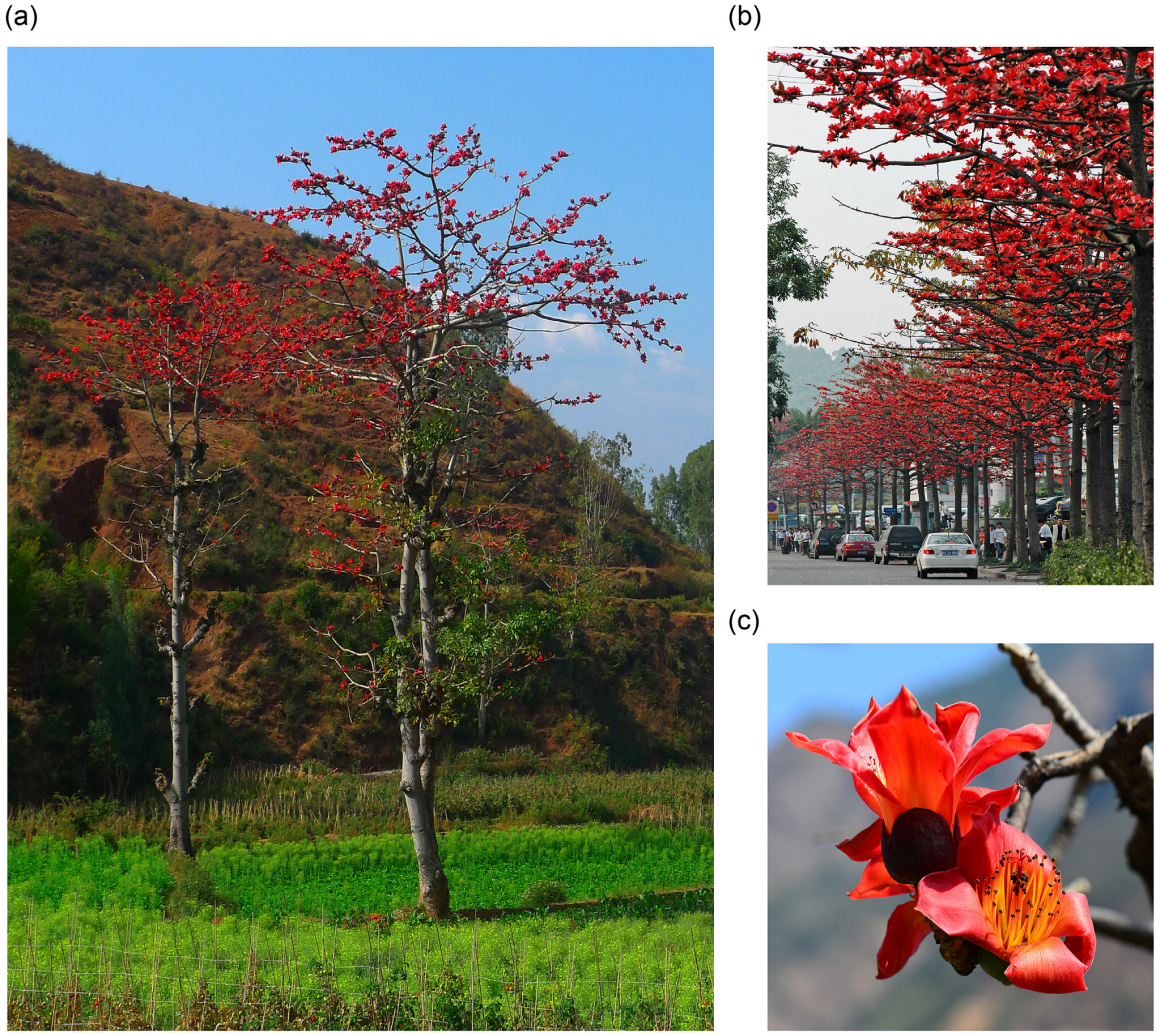
Example of the red silk cotton tree (*B. ceiba*). A) Natural habitat of *B. ceiba* (image from Guanglong Ou). B) *B. ceiba* used as municipal greening trees (image from Jianmei Wu). C) The flower of *B. ceiba* (image from Renbin Zhu).

*Bombax ceiba* Linn. (Malvaceae), commonly known as the cotton tree or red silk cotton tree, is a spectacular flowering tree with a height of up to 40 meters (Fig. 1a) that is found in tropical and sub-tropical Asia as well as northern Australia [1]. It has been chosen as the “city flower” of the cities of Kaohsiung and Guangzhou for its large, showy flowers with thick, waxy, red petals that densely clothe leafless branch tips in late winter and early spring (Fig. 1b, c). *B. ceiba* is a source of food, fodder, fiber, fuel, medicine, and many other valuable goods for natives of many Asian countries [2]. For example, its fruits are good sources of silk-cotton for making mattresses, cushions, pillows, and quilts [3], while its timbers are widely used in matches, boxes, and splints [4]. Moreover, studies on the cotton tree have shown that it produces many novel secondary metabolites and have explored its traditional medicinal usage by various tribal communities [1, 2, 5, 6]. In addition to its economic and medicinal value, *B. ceiba* is an ecologically important plant: it is a reforestation pioneer that survives easily in low-rainfall and well-drained conditions [7] and has been identified as a plant species suitable for municipal greening because of its capacity to counteract the detrimental effects of air pollution [8, 9].

Despite the considerable economic and ecological importance of *B. ceiba*, the genomic information available for this species is limited, which has hindered its utilization. Here we report a draft genome sequence for *B. ceiba* that is expected to facilitate and expand its use.

### Sampling and sequencing

All samples were collected from Yuanmou, Yunnan Province, China (25°40′50.06″ N, 101°53′27.76″ E). Genomic DNA was extracted from leaves of a single tree using the Plant Genomic DNA kit (Tiangen, Beijing, China). A SMRTbell DNA library was then prepared and sequenced using P6, C4 chemistry according to the manufacturer's protocols (Pacific Biosciences, CA, USA), and a 20-kb SMRTbell library was generated using a BluePippin DNA size selection instrument (Sage Science, MA, USA) with a lower size limit of 10 kb. Single-molecule real-time sequencing of long reads was conducted on a PacBio Sequel platform with 19 SMRT cells. A total of 86.0 Gb of genomic data with an average read length of 8.4 kbwas generated after quality filtering (Supplementary Table S1). In addition, a separate paired-end (PE) DNA library with an insert size of 400 bp (amplification by eight PCR cycles) was constructed and sequenced using the Illumina platform (PE 150) to enable a genome survey. The NGS sequencing produced 36.1 Gb of raw data, of which 20.0 Gb remained after filtering.

Total RNA was extracted from the bud, root, bark, flower, and fruit tissues of one *B. ceiba* individual using the QIAGEN RNeasy Plant Mini Kit (QIAGEN, Hilden, Germany). RNA-seq libraries were then prepared using the TruSeq RNA Library Preparation Kit (Illumina, CA, USA), and paired-end sequencing with a read length of 150 bp was conducted on the HiSeq 2000 platform, yielding 44.41 Gb of clean data (30,816,034−51,191,192 reads per sample) (Supplementary Table S2).

### Genome size and heterozygosity estimation

The genome size of *B. ceiba* was estimated by the K-mer method [10] using sequencing data from the Illumina DNA library. Quality-filtered reads were subjected to 17-mer frequency distribution analysis using the Jellyfish program [10]. The count distribution of 17-mers followed a Poisson distribution, with the highest peak occurring at a depth of 22 (Supplementary Table S3 and Fig. S1). The estimated genome size was approximately 809,166,127 bp, and the heterozygosity rate of the *B. ceiba* genome was approximately 0.88%.

### Genome assembly

Genome assembly was performed on full PacBio long reads using FALCON v0.3.0 [11]. Error correction and pre-assembly were carried out with the FALCON pipeline after evaluating the outcomes of using different parameters in FALCON during the pre-assembly process. Based on the contig N50 results, a length_cutoff of 11kb and a length_cutoff_pr of 11.5kb for the assembly step were ultimately chosen. The draft assembly was polished using Arrow [12], which mapped the PacBio reads to the assembled genome with the Blasr pipline [13]. The preliminary genome assembly was approximately 852 Mb in size, with a contig N50 size of 727 kb. A GC depth analysis was conducted to assess the potential contamination during sequencing and the coverage of the assembly, revealing that the genome had an average GC content of 33.3% and a unimodal GC content distribution (Supplementary Fig. S2). The GC depth as well as the sequencing depth of the genome assembly suggested that there was no contamination from other species (Supplementary Fig. S3). To further assess contaminations, we searched all sequences of the genome assembly against the NCBI non-redundant nucleotide database (Nt) with BLASTN [14] (E-value ≤ 1e−5). In total, 2,494 significant hits were achieved. The top-hit species were *Theobroma cacao* and *Gossypium* species, comprising more than 69% ( 1,733 hits) of the hits (Supplementary Table S4). Only five hits from four non-plant species (*Psyllidae* sp., *Trioza eugeniae, Diptacus* sp., and *Dichorragia nesimachus*) were detected (Supplementary Table S4), suggesting there was no potential contamination from non-plant species in the genome of *Bombax ceiba*.

### Scaffolding with BioNano optical mapping

The purified genomic DNA of *B. ceiba* was embedded in an agarose layer, digested with Nt. BspQI enzyme, and labeled. The molecules were counterstained using the protocol provided with the IrysPrep Reagent Kit (BioNano Genomics, San Diego, USA). Samples were then loaded into IrysChips and imaged on an Irys imaging instrument (BioNano Genomics, San Diego, USA). After filtering using a molecule length cutoff of <150kb, a molecule SNR of <2.75, a label SNR of <2.75, and a label intensity of >0.8, 160.0 Gb of BioNano clean data were obtained, with the N50 size of the labeled single molecules being 269.9 kb (Supplementary Table S5).

A molecular quality report was generated by aligning the BioNano library sequences to the initial PacBio genome assembly, yielding a map rate of 34.2%. Using the PacBio genome assembly data as a reference, a reference genome assembly was conducted based on the clean BioNano data. A genome map consisting of 2,023 consensus maps was assembled, yielding a genome size of 1.09 Gb with an N50 size of 0.7 Mb. The average molecule coverage depth of the genome map was about 27 folds. To obtain a longer scaffold, the *de novo* assembly of PacBio reads was then mapped to the BioNano single-molecule genomic map. After scaffolding, the contig assembly contained 3,105 scaffolds with a scaffold N50 of 1.5 Mb.

To fill the gaps in the scaffolds, the Blasr pipline [13] was used to map the PacBio long reads to the draft genome assembly scaffolding with BioNano optical mapping. The draft was polished using PBJelly 2 (PBJelly, RRID:SCR_012091) [15] over three iterations. Reads from the Illumina DNA library (400bp) were then aligned against the genome assembly using the BWA software (BWA, RRID:SCR_010910) to fill the gaps and correct potential sequencing errors of the assembly, and a mapping rate of 99.2% was achieved [16]. The final assembly was polished using Pilon [17], yielding a final draft genome of approximately 895 Mb, with contig and scaffold N50 sizes of 1.0 Mb and 2.06 Mb, respectively (SupplementaryTable S6).

### Evaluation of the completeness of the genome assembly gene space

To evaluate the coverage of the assembly, we aligned all the RNA-seq reads against the *B. ceiba* genome assembly using HISAT [18] with default parameters. The percentage of aligned reads ranged from 84.78% to 91.08% (Supplementary Table S2). We then used Benchmarking Universal Single-Copy Orthologs (BUSCO, RRID:SCR_015008) [19] to search the annotated genes in the assembly for the 1,440 single-copy genes conserved among all embryophytes. About 94.4% of the complete BUSCOs were found in the assembly (Supplementary Table S7). These results suggested that the genome assembly was complete and robust.

### Genome annotation

The repeat sequences in the genome consisted of simple sequence repeats (SSRs), moderately repetitive sequences, and highly repetitive sequences. The MISA tool [20] was used to search for SSR motifs in the *B. ceiba* genome, with default parameters. A total of 454,435 SSRs were identified in this way: 310,369, 105,004, 30,925, 6,448, 1,165,and 524 mono-, di-, tri-, tetra-, penta-, and hexa-nucleotide repeats, respectively (Supplementary Table S8).

To identify known transposable elements (TEs) in the *B. ceiba* genome, RepeatMasker (RepeatMasker, RRID:SCR_012954) [21] was used to screen the assembled genome against the Repbase (v. 22.11) [22] and Mips-REdat libraries [23]. In addition, *de novo* evolved annotation was performed using RepeatModeler v. 1.0.11 (RepeatModeler, RRID:SCR_015027) [21]. The combined results of the homology-based and *de novo* predictions indicated that repeated sequences account for 60.30% of the *B. ceiba* genome assembly (Supplementary Table S9), with long terminal repeats accounting for the greatest proportion (47.86%) (Supplementary Table S9).

Homology-based ncRNA annotation was performed by mapping plant rRNA, miRNA, and snRNA genes from the Rfam database (release 13.0) [24] to the *B. ceiba* genome using BLASTN [14] (E-value ≤ 1e−5). tRNAscan-SE v1.3.1 (tRNAscan-SE, RRID:SCR_010835) [25] was used (with default parameters for eukaryotes) for tRNA annotation. RNAmmer v1.2 [26] was used to predict rRNAs and their subunits. These analyses identified 496 miRNAs, 894 tRNAs, 6,772 rRNAs, and 727 snRNAs (Supplementary Table S10).

The homology-based and *de novo* predictions were also used to annotate protein coding genes. For homology-based predictions, protein sequences from four species (*Arabidopsis thaliana, Carica papaya, Gossypium arboretum*, and *T. cacao*) (Supplementary Table S11) were mapped onto the *B. ceiba* genome; the aligned sequences and the corresponding query proteins were then filtered and passed to GeneWise v2.4.1 (GeneWise, RRID:SCR_015054) [27] to search for accurately spliced alignments. For the *de novo* predictions, we first randomly selected 1,000 full-length genes from the homology-based predictions to train model parameters for Augustus v3.0 (Augustus: Gene Prediction, RRID:SCR_008417) [28], GeneID v1.4.4 [29], GlimmerHMM (GlimmerHMM, RRID:SCR_002654) [30], and SNAP [31]. Augustus v3.0 [28], GeneID v1.4.4 [29], GlimmerHMM [30], and SNAP [31] were then used to predict genes based on the training set. Finally, EVidenceModeler v1.1.1 [32] was used to integrate the predicted genes and generate a consensus gene set (Supplementary Table S11). Genes with TEs were discarded using the TransposonPSI [33] package. Low-quality genes consisting of fewer than 50 amino acids and/or exhibiting premature termination were also removed from the gene set, yielding a final set of 52,705 genes. The final set's average transcript length, average CDS length, and exon number per gene were 2,418.37 bp, 1,019.38 bp, and 4.57, respectively (Supplementary Table S12 and Fig. S4).

The annotations of the predicted genes of *B. ceiba* were screened for homology against the Uniprot (release 2017_10) and KEGG (release 84.0) databases using Blastall [14] and KAAS [34]. Then, the InterProScan (InterProScan, RRID:SCR_005829) [35] package was used to annotate the predicted genes using the InterPro (5.21–60.0) database. In total, 47,105 of the total 52,705 genes (89.37 %) were annotated with potential functions (Supplementary Table S13).

### Phylogenetic tree construction and divergence time estimation

**Figure 2:**
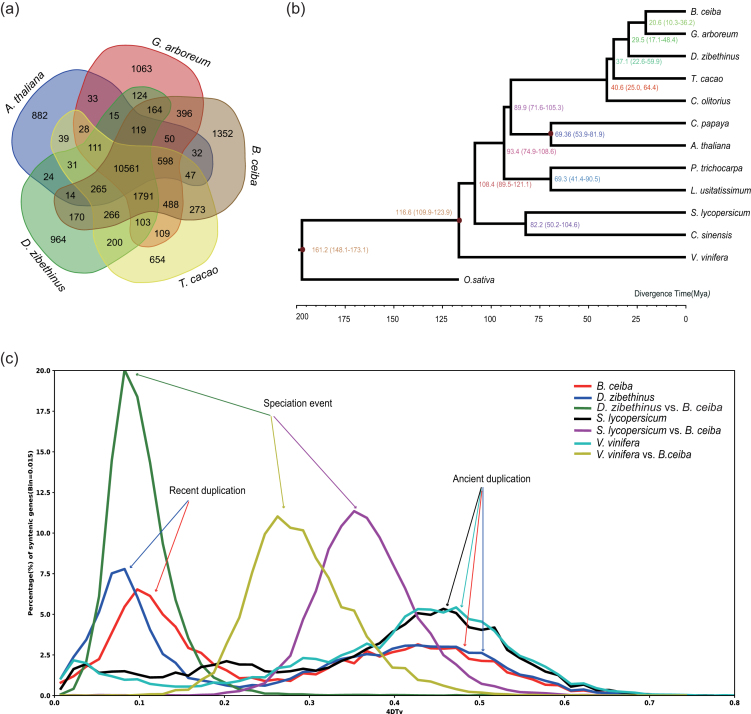
Phylogenetic relationships and genomic comparisons between *B. ceiba* and other plants. A) A Venn diagram of shared gene families between *B. ceiba* and three other Malvales plants, with *A. thaliana* as an outgroup. Each number represents a gene family number. B) Inferred phylogenetic tree across 13 plant species. The estimated divergence time (Mya) is shown at each node. C) Whole-genome duplication (WGD) events of four plants (*B. ceiba, D. zibethinus, S. lycopersicum*, and *V. vinifera*) inferred by four-fold synonymous third-codon transversion (4DTv) estimations. Peaks corresponding to speciation, recent, and ancient WGDs are indicated by arrows.

To investigate the evolutionary position of *B. ceiba*, we compared its genome to the genome sequences of 12 other plants. These included four plants in the Malvales order (*Gossypium arboreum, Durio zibethinus, Corchorus olitorius*, and *T. cacao*), seven plants from different orders in the same Eudicots clade (*Arabidopsis thaliana, Carica papaya, Linum usitatissimum, Populus trichocarpa, Camellia sinensis, Solanum lycopersicum*, and *Vitis vinifera*), and *Oryza sativa* as an outgroup. Genome sequences from *A. thaliana, T. cacao, C. papaya, L. usitatissimum, P. trichocarpa, C. sinensis, S. lycopersicum, V. vinifera*, and *O. sativa* were downloaded from Phytozome v. 12.0 [36]. Gene sequences of *G. arboreum, C. olitorius*, and *D. zibethinus* were downloaded from the NCBI Database (PRJNA335838, PRJNA215141 and PRJNA400310). We used the OrthoMCL (v2.0.9) pipeline (OrthoMCL DB: Ortholog Groups of Protein Sequences, RRID:SCR_007839) [37] (BLASTP E-value ≤ 1e−5) to identify potentially orthologous gene families within these genomes. Gene family clustering identified 16,586 gene families containing 37,736 genes in *B. ceiba* (Fig. 2a). Of these, 906 gene families were unique to *B. ceiba* (Supplementary Table S14). *B. ceiba* and other Malvales plants had the largest number of shared gene families among the studied plants.

Phylogenetic analysis was performed using 172 single-copy orthologous genes from common gene families found by OrthoMCL [37] (Supplementary Fig. S5). We codon-aligned each gene family using MUSCLE (MUSCLE, RRID:SCR_011812) [38] and curated the alignments with Gblocks v0.91b [39]. Phylogeny analysis was performed using RAxML (RAxML, RRID:SCR_006086) v 8.2.11 [40] with the GTRGAMMA model and 100 bootstrap replicates. We then used MCMCTREE as implemented in PAML v4.9e (PAML, RRID:SCR_014932) [41] to estimate the divergence times of *B. ceiba* from the other plants. The parameter settings of MCMCTREE were as follows: clock = 2, RootAge ≤ 1.73, model = 7, BDparas = 110, kappa_gamma = 62, alpha_gamma = 11, rgene_gamma = 23.18, and sigma2_gamma = 14.5. In addition, the divergence times of *O. sativa* (148–173 Mya), *V. vinifera* (110–124 Mya),and *A. thaliana* (53–82 Mya) were used for fossil calibration. The phylogenetic analysis showed that *B. ceiba* is more closely related to *G. arboraum* than to *D. zibethinus* (Supplementary Fig. S6), which supports the well-established hypothesis of a close relationship between Bombacaceae and Malvaceae [42, 43]. Recent phylogenetic studies have suggested that the group traditionally referred to as Bombacaceae (which includes the tribe Durioneae) is not actually monophyletic, and that the genera of the tribe Durioneae should be excluded from Bombacaceae. Most members of the erstwhile family Bombacaceae have been transferred to the subfamily Bombacoideae within the family Malvaceae [43]. This phylogenetic ordering was supported by our phylogenetic analysis of the complete chloroplast genomes of Marvel plants [44]. The estimated divergence time of *B. ceiba* and *D. zibethinus* was 29.5 Mya, while that of *B. ceiba* and *G. arboretum* was about 20.6 Mya (Fig. 2b).

### Genes under positive selection

*B. ceiba* is an ecologically important plant that could survive in extreme climate conditions, such as hot-dry valleys [7]. According to the neutral theory of molecular evolution [45], the ratio of nonsynonymous substitution rate (Ka) and synonymous substitution rate (Ks) of protein coding genes can be used to identify genes that show signatures of natural selection. We calculated average Ka/Ks values and conducted the branch-site likelihood ratio test using Codeml implemented in the PAML package [41] to identify positively selected genes in the *B. ceiba* lineage. These genes might contribute to the adaption to unfavorable environments. Thirty-six genes with signatures of positive selection were identified (*P* ≤ 0.05), of which 32 genes could be annotated with potential functions in the Swissport database (Supplementary Table S15). One gene is homologous to a desiccation protectant protein coding gene (*Lea14*). There is a strong association of LEA proteins with abiotic stress tolerance, particularly during dehydration and cold stress [46]. This gene could potentially contribute to the adaption of *B. ceiba* to the dry valley environment. Another gene showing signs of positive selection is homologous to the gene coding for Kelch domain-containing protein 4. The Kelch domain-containing proteins are involved in regulating major processes such as growth, development, and biotic and abiotic stress responses in plants [47, 48]. Some researchers suggested that the E3 ubiquitin-protein ligase (RFWD3) has potential roles in plant stress responses [49, 50]. Twenty-one positively selected sites were identified in the CACTIN protein coding gene. The CACTIN protein was characterized as a negative regulator of many different developmental processes, such as embryogenesis [51]. While literature reports are rare, other identified genes might also be associated with the ecological adaption of *B. ceiba*. It should be noted that this is just a preliminary analysis of the functions of these genes; further studies would be needed to clarify their roles.

### Whole-genome duplication and gene family expansion analysis

We used 4DTv estimation to detect WGD events in the *B. ceiba* genome. To this end, paralogous sequences of *B. ceiba, T. cacao, V. vinfira, S. lycopersicum*, and *D. zibethinus* was identified with OrthoMCL [37]. Then, protein sequences for each of these plants were aligned against each other with Blastp [14] (using an E-value threshold of ≤1e−5) to identify conserved paralogs in each species. Finally, potential WGD events in each genome were evaluated based on their 4DTv distribution. The WGD analysis suggested that *B. ceiba* experienced the same WGD events as other Dicotyledons, and that *B. ceiba* and *D. zibethinus* went through their WGD events before diverging from their common ancestor (Fig. 2c).

The OrthoMCL gene family analysis results were analyzed further by using Computational Analysis of Gene Family Evolution, v3.0 [52] to detect expanded gene families. This approach revealed 5,612 expanded gene families and 1,902 contracted gene families in the *B. ceiba* lineage (Supplementary Fig. S7).

## Conclusion

This paper reports the sequencing, assembly, and annotation of the *B. ceiba* genome along with details of its evolutionary history. The genomic data generated in this work will be a valuable resource for further genetic improvement and effective use of the red silk cotton tree.

## Availability of supporting data

The raw data from our genome project was deposited in the SRA (Sequence Read Archive) database of National Center for Biotechnology Information with Bioproject ID PRJNA429932. The assembly and annotation of the *B. ceiba* genome and other supporting data, including BUSCO results, are available in the *GigaScience* database, GigaDB [53]. Versions and main parameters of the software used in this study are provided in Supplementary Table S16.

## Additional files

**Figure S1**. Frequency distribution of the 17-mer graph analysis used to estimate the size of the *B. ceiba* genome.

**Figure S2**. GC content distribution of the *B. ceiba* genome. The GC content was established using 500-bp slidingwindows.

**Figure S3**. The GC depth distribution of the *B. ceiba* genome.

**Figure S4**. Comparison of gene structure characteristics in *B. ceiba* to that in other plants. a, CDS length; b, exon length; c, exon number; d, gene length; e, intron length.

**Figure S5**. Gene orthology determined by comparing genomes using the OrthoMCL software.

**Figure S6**. The maximum-likelihood phylogeny of *B. ceiba* and 13 other plants.

**Figure S7**. Gene family expansions and contractions in *B. ceiba* and 13 other plants.

**Table S1**. Sequencing statistics from the PacBio platform.

**Table S2**. Summary of the transcriptomes and their mapping rates on the genome assembly.

**Table S3**. Estimation of genome size based on 17-mer statistics.

**Table S4**. Blast results of *B. ceiba* genome against the NCBI Nt database.

**Table S5**. Summary of the BioNano optical mapping data.

**Table S6**. Summary of the final genome assembly.

**Table S7**. Summary of BUSCO analysis results.

**Table S8**. Summary of the SSR search results.

**Table S9**. Repeat annotation of the *B. ceiba* genome assembly.

**Table S10**. Summary of non-protein-coding gene annotations in the *B. ceiba* genome assembly.

**Table S11**. Gene annotation statistics of the *B. ceiba* genome assembly.

**Table S12**. Comparative gene statistics.

**Table S13**. Functional annotation of predicted genes of *B. ceiba*.

**Table S14**. Summary statistics of gene families in 13 plant species.

**Table S15**. Candidate positively selected genes in the *B. ceiba* lineage.

**Table S16**. Versions and main parameters of the software used in this study.

## Abbreviations

SMRT: single molecule real time; 4DTv: four-fold synonymous third-codon transversion.

## Competing interests

S.S. is an employee of Nextomics Bioscences. Other authors declare that they have no competing interests.

## Authors’ contributions

L.T. and B.T. designed the project; H.W., C.L., and H.C. collected samples and extracted the DNA and RNA samples; Y.G., S.S., H.,W. and C.L. worked on sequencing and data analyzing; Y.G. wrote the manuscript; L.T., B.T., and D.D. revised the manuscript; Y. G., H. W., C. L., H. C., D. D., S. S., L. Y., L. H., Y. F., B. T. and L. T. read and approved the final version of the manuscript.

## Supplementary Material

GIGA-D-18-00045_Original_Submission.pdf

GIGA-D-18-00045_Revision_1.pdf

GIGA-D-18-00045_Revision_2.pdf

Response_to_Reviewer_Comments_Original_Submission.pdf

Response_to_Reviewer_Comments_Revision_1.pdf

Reviewer_1_Report_(Original_Submission) -- Nicolas Delhomme, Ph. D. rer. nat. 2/23/2018 Reviewed

Reviewer_2_Report_(Original_Submission) -- Yuxuan Yuan 2/27/2018 Reviewed

Reviewer_2_Report_(Revision_1) -- Yuxuan Yuan 3/19/2018 Reviewed

Reviewer_2_Report_(Revision_2) -- Yuxuan Yuan 4/8/2018 Reviewed

Supplemental material
