# Supplementary material for: De novo genome assembly of the red silk cotton tree (Bombax ceiba)
Source: Gigascience. 2018 May 10;7(5):giy051. doi: 10.1093/gigascience/giy051 (PMC5967522; doi:10.1093/gigascience/giy051)
Supplement: GIGA-D-18-00045_Revision_1.pdf [file giy051_giga-d-18-00045_revision_1.pdf]

GigaScience

De novo genome assembly of the red silk cotton tree (Bombax ceiba)

--Manuscript Draft--

**Manuscript Number:**	GIGA-D-18-00045R1
**Full Title:**	De novo genome assembly of the red silk cotton tree (Bombax ceiba)
**Article Type:**	Data Note
**Funding Information:**	National Natural Science Foundation of China (31460561) — Dr. Lizhou Tang<br>National Natural Science Foundation of China (31760103) — Dr. Yong Gao<br>National Natural Science Foundation of China (31460179) — Dr. Haibo Wang<br>National Natural Science Foundation of China (31660680) — Dr. Long Yu<br>Applied Basic Research Key Project of Yunnan (2017FD145) — Dr. Yong Gao
**Abstract:**	Background: Bombax ceiba L. (the red silk cotton tree) is a large deciduous tree that is distributed in tropical and sub-tropical Asia, and northern Australia. It has great economic and ecological importance, with several applications in industry and traditional medicine in many Asian countries. To facilitate the further utilization of this plant resource, we present here the draft genome sequence for B. ceiba.<br>Findings: We assembled a relatively intact genome of B. ceiba by using PacBio single-molecule sequencing and BioNano optical mapping technologies. The final draft genome is approximately 895 Mb long, with contig and scaffold N50 sizes of 1.0 Mb and 2.06 Mb, respectively.<br>Conclusions: The high-quality draft genome assembly of B. ceiba will be a valuable resource enabling further genetic improvement and more effective use of this tree species.
**Corresponding Author:**	Lizhou Tang<br>Center for Yunnan Plateau Biological Resources Protection and Utilization<br>Qujing, Yunnan CHINA
**Corresponding Author Secondary Information:**	
**Corresponding Author's Institution:**	Center for Yunnan Plateau Biological Resources Protection and Utilization
**Corresponding Author's Secondary Institution:**	
**First Author:**	Yong Gao
**First Author Secondary Information:**	
**Order of Authors:**	Yong Gao<br>Haibo Wang<br>Chao Liu<br>Honglong Chu<br>Dongqin Dai<br>Shengnan Song<br>Long Yu<br>Lihong Han

Powered by Editorial Manager® and ProduXion Manager® from Aries Systems Corporation

	Yi Fu
	Bin Tian
	Lizhou Tang
**Order of Authors Secondary Information:**	
**Response to Reviewers:**	Dear editors and reviewers,<br>The manuscript "De novo genome assembly of the red silk cotton tree (Bombax ceiba)" (GIGA-D-18-00045) has been carefully revised. The major revisions are marked in red, and the language has been polished by "sees-editing Ltd". We appreciate the detailed, useful comments and suggestions from you and the reviewers.<br>As the editor suggested,<br>1. The paper is submitted as a Data Note, and as such we do not require in-depth biological analyses. However, I do agree with reviewer 2 (their point #4), that some information on important genes and pathways will help to demonstrate the usefulness of the dataset.<br>Answer: As the editor and the reviewer suggested, information on important genes and pathways will help to demonstrate the usefulness of our genome data. B. ceiba is an ecologically important plant which could survive in extreme climate conditions. We calculated the average Ka/Ks values and conducted a branch-site likelihood ratio test to identify positively selected genes which could contribute to the ecological adaptation of B. ceiba. Thirty-six genes were identified, and some annotated genes were reported to be associated with ecological adaption. Please see line 190 to 210 in the revised manuscript.<br>2. Regarding minor point # 3 of reviewer 2: " L177: It may not be good to say this. Please rewrite. " I am actually not quite sure myself what the reviewer refers to here, but if I understand correctly, they feel the wording in that line 177 comes across as a bit cumbersome; this is true, but I'd prefer to have as much detail on parameters etc in the methods section as possible, and you should definitely keep that useful information.<br>Answer: As the editor suggested, the information on parameters was very important. So we kept this information in the revised manuscript.<br>3. Another minor remark: In the introduction, you refer to the use in Traditional Chinese Medicine: "Moreover, studies ... have validated its traditional medicinal usage." As this is still an active area of research, I feel it would be more appropriate to write "studies.... have explored its traditional medicine usage" instead of "validated".<br>Answer: The suggestion has been taken seriously, and this sentence has been changed to "studies.... have explored its traditional medicine usage". Please check line 41.<br><br>The point by point answers to the comments and suggestions of the reviewers are below.<br>Reviewer: 1<br>1. For every software, please (if not in the manuscript, then in the supplementary text) provide the non default parameters that were used, if any.<br>Answer: The suggestion has been taken seriously, and the versions and parameters of software used in our study have been provided as one supplementary table. Please see Table S15 in the supplementary file.<br>2. Line 60 precise the type (SE, PE, MP) and number of cycle of the Illumina sequencing.<br>Answer: We accept the review's suggestion, and the sequencing strategy (PE) has been added. Please see line 60 in the revised manuscript.<br>3. Line 106 - I would like some more details about the Illumina based scaffolding. Given that it's a small insert size library (400bp), how helpful was it to scaffold the PacBio assembly. Or do you mean the RNA-Seq data was used to scaffold? If that's the case, what parameters were used and how were multiple mapping case handled?<br>Answer: The sequencing data from the Illumina DNA library (400bp) was aligned against the genome assembly. As the reviewer stated, the small insert size library had relatively limited effects on scaffolding. The analysis was aimed at filling gaps and correcting some sequencing errors of the genome assembly. Sorry for the ambiguity. The sentence has been corrected. Please see line 106 to 108.<br>4. Line 111 - I would replace the title there to: Evaluation of the completeness of the genome assembly gene space. BUSCO only look at the gene space. For the completeness of the genome assembly, one would need different metrics, such as FRC (feature response curve) or any quantification of the reads that do not align to the

final assembly.
Answer: We take the review's suggestion, and the title has been changed to "Evaluation of the completeness of the genome assembly gene space".
5. Line 226 - There's a typesetting error in "financially"
Answer: We accepted the review's suggestion, and this error has been corrected in the revised manuscript.

Discretionary comments
1. Line 114 - Have you analysed the reads that do not align to the genome, what are these?
Answer: Unfortunately, we did not analyze the RNA reads which could not align to the genome assembly, and we did not know the details on these reads.
2. Line 116 - Have you checked what the 5.6% of incomplete BUSCO genes are?
Answer: There are 63 missing BUSCO genes and 19 incomplete BUSCO genes in total. We have searched these genes against the OrthoDB database to infer the gene functions.
For the 63 missing genes, there are eight genes falling in the category of pentatricopeptide repeat (PPR) superfamily protein, seven genes belonging to protein kinase domain, six genes annotated as transmembrane: Helical, five genes belonging to the tetratricopeptide repeat (TPR)-like superfamily proteins, and five genes annotated with unknown functions. The rest of genes were annotated with a variety of functions, such as peptidyl-prolyl cis-trans isomerase, WD40 repeat, regulator of chromosome condensation (RCC1) family protein, and so on.
For the 19 incomplete genes, two genes were characterized as transmembrane: Helical, and two genes were annotated with unknown functions. The other genes were annotated with a variety of functions, such as Zinc finger (C2H2), Reticulon-like protein, and so on.
3. Line 152 - The annotation could be refined using PASA. This would add splice variants to the annotation. Also, the PASA output can be post-processed to identify lncRNA. Many tools now allow for an in-depth analysis of differential transcript expression, differential transcript usage, etc. leveraging from more complete annotations.
Answer: As the reviewer suggested, PASA was a useful tool, especially for annotating splice variants. And we will try this software in our future study.

Reviewer: 2
1. Authors used a Kmer-based method to estimate the genome size. However, it seems the estimated genome size is smaller than their assembly size. Do authors know what happens here?
Answer: As the reviewer pointed, there was some inconstancy between the size of the genome survey and the genome assembly. As the heterozygosity rate of the B. ceiba genome was 0.88%, heterozygous regions which could not be assembled into consensus sequences might result in larger assembly. Besides, the Kmer-based method was just a preliminary estimation of the genome size. There could be some small deviation from the genome assembly.
2. For contamination checking, do authors believe a visualised GC content plot and the average GC content can tell there is no contamination in the assembly? Why didn't authors use other methods to check, such as BLASTN?
Answer: There were two strategies for deducing contaminations by GC content. 1. There were uneven GC contents in the genome. 2. The GC content was constant, but the sequencing depth varied among sequences. BLASTN was also a useful method for assessing contaminations. The suggestion of the reviewer has been taken seriously, and we randomly selected some sequences from the genome assembly and searched the data against the NCBI database of bacteria with BLASTN. No contamination from bacteria was detected. Please check line 88 to 91 in the manuscript.
3. From the report, there is a ~150Mb difference between the BioNano consensus maps and the PacBio assembly. Does that mean there are repeat collapses in the PacBio assembly or some genome regions cannot be covered by PacBio reads? How does it look like in the comparison between BioNano consensus maps and the PacBio assembly? Do they align well?
Answer: The BioNano consensus genome map recorded the order of sequence fragments and the gap size between adjacent contigs. The BioNano genome map included gaps (Ns), whereas contigs of the primary PacBio assembly did not contain any gaps. It might be the reason why there was a ~150Mb difference. And the

Powered by Editorial Manager® and ProduXion Manager® from Aries Systems Corporation

	inconstancy between BioNano genome map and genome assembly size was also observed in other species, such as Fragaria vesca [1] and Tribolium castaneum [2]. With the initial PacBio genome assembly as reference, approximately 54 Gb out of 160 Gb BioNano clean data could be assigned to the genome map, and the effective coverage of assembly was about 60 X.<br><br>Reference<br>1.Edger PP, VanBuren R, Colle M, et al. Single-Molecule Sequencing and Optical Mapping Yields an Improved Genome of Woodland Strawberry (Fragaria Vesca) with Chromosome-Scale Contiguity. GigaScience. 2018;7 (2):1-7. doi:10.1093/gigascience/gix124.<br>2.Shelton JM, Coleman MC, Herndon N, et al. Tools and Pipelines for Bionano Data: Molecule Assembly Pipeline and Fasta Super Scaffolding Tool. BMC Genomics. 2015;16 (1):734.<br><br>4. This research is more likely a technical and bioinformatics report. Some biological stories, such as important genes, their functions and roles in certain pathways, are missing. How can this research help to improve Bombax ceiba's economic and ecological values?<br>Answer: As the reviewer suggested, information on important genes and pathways will help to demonstrate the usefulness of our genome data. B. ceiba is an ecologically important plant which could survive in extreme climate conditions. So we calculated the average Ka/Ks values and conducted a branch-site likelihood ratio test to identify positively selected genes which could contribute to the ecological adaptation of B. ceiba. Thirty-six genes were identified, and some annotated genes were reported to be associated with ecological adaption. Please see line 190 to 210 in the revised manuscript.<br>5. The English in the manuscript needs a further edit<br>Answer: We accepted the reviewer’s suggestion, and the language has been polished by “sees-editing Ltd”.<br>Minor:<br>1. It would be good to specify the version of each tool and detail the parameter settings.<br>Answer: We accepted the reviewer’s suggestion, and the versions and parameters of software used in our manuscript were provided as one supplementary table. Please see Table S15 in the supplementary file.<br>2. It would be good to map the Illumina short reads (DNA) back to the assembly and give a mapping rate to inform the quality of the assembly.<br>Answer: We accepted the reviewer’s suggestion, and we have mapped the Illumina short reads (DNA) back to the assembly. A mapping rate of 99.2% was achieved Please check line 106 to 108 in the revised manuscript.<br>3. L177: It may not be good to say this. Please rewrite.<br>Answer: As the reviewer suggested, we rewrote part of the sentence. As the detail on parameters was very important for this analysis, we kept this information in the revised manuscript. Please check line 178 in the manuscript.<br>4. FigureS4 and TableS4 are not informative. May change them to a table to list the stats of the raw BioNano maps.<br>Answer: We took the reviewer’s suggestion, and FigureS4 was removed. TableS4 was changed to a table which included statistics of the raw BioNano maps.<br>5. TableS5 may add two more columns: one is for the PacBio assembly, and the other one is for the BioNano optical scaffolding. From the improved table, readers can easily tell the changes before and after BioNano scaffolding.<br>Answer: We took the reviewer’s suggestion, and this information was added to TableS5. Please check Table S5 in the supplementary file.<br><br>Finally, we appreciate much your time in editing our manuscript and the reviewers for their valuable suggestions and comments. We look forward to hear your final decision when it is made.
**Additional Information:**	
**Question**	**Response**
Are you submitting this manuscript to a special series or article collection?	No

Powered by Editorial Manager® and ProduXion Manager® from Aries Systems Corporation

**Experimental design and statistics**<br><br>Full details of the experimental design and statistical methods used should be given in the Methods section, as detailed in our Minimum Standards Reporting Checklist. Information essential to interpreting the data presented should be made available in the figure legends.<br><br>Have you included all the information requested in your manuscript?	Yes
**Resources**<br><br>A description of all resources used, including antibodies, cell lines, animals and software tools, with enough information to allow them to be uniquely identified, should be included in the Methods section. Authors are strongly encouraged to cite Research Resource Identifiers (RRIDs) for antibodies, model organisms and tools, where possible.<br><br>Have you included the information requested as detailed in our Minimum Standards Reporting Checklist?	Yes
**Availability of data and materials**<br><br>All datasets and code on which the conclusions of the paper rely must be either included in your submission or deposited in publicly available repositories (where available and ethically appropriate), referencing such data using a unique identifier in the references and in the "Availability of Data and Materials" section of your manuscript.<br><br>Have you have met the above requirement as detailed in our Minimum Standards Reporting Checklist?	Yes

Manuscript

Click here to download Manuscript 20180312De novo genome assembly of the red silk cotton tree.docx

De novo genome assembly of the red silk cotton tree (*Bombax ceiba*)

Yong Gao[1, #], Haibo Wang[1, #], Chao Liu[1, #], Honglong Chu[1], Dongqin Dai[1], Shengnan Song[5], Long Yu[1], Lihong Han[1], Yi Fu[2], Bin Tian[2,3, *], Lizhou Tang[1,4, *]

[1] Center for Yunnan Plateau Biological Resources Protection and Utilization, College of Biological Resource and Food Engineering, Qujing Normal University, Qujing, Yunnan, 655011, China

[2] Key Laboratory of Biodiversity Conservation in Southwest China, State Forestry Administration, Southwest Forestry University, Kunming 650224, China

[3] Key Laboratory of Biodiversity and Biogeography, Kunming Institute of Botany, Chinese Academy of Sciences, Kunming 650204, China

[4] State Key Laboratory of Genetic Resources and Evolution，Kunming Institute of Zoology，Chinese Academy of Sciences, Kunming 650223, China

[5] Nextomics Biosciences Institute, Wuhan, Hubei 430000, China

[#] These authors contributed equally to this work.

[*] Correspondence should be addressed to Lizhou Tang (tanglizhou@163.com) and Bin Tian (tianbinlzu@163.com).

De novo genome assembly of the red silk cotton tree (_Bombax ceiba_)

Abstract

Background: *Bombax ceiba* L. (the red silk cotton tree) is a large deciduous tree that is distributed in tropical and sub-tropical Asia, and northern Australia. It has great economic and ecological importance, with several applications in industry and traditional medicine in many Asian countries. To facilitate the further utilization of this plant resource, we present here the draft genome sequence for *B. ceiba*.

Findings: We assembled a relatively intact genome of *B. ceiba* by using PacBio single-molecule sequencing and BioNano optical mapping technologies. The final draft genome is approximately 895 Mb long, with contig and scaffold N50 sizes of 1.0 Mb and 2.06 Mb, respectively.

Conclusions: The high-quality draft genome assembly of *B. ceiba* will be a valuable resource enabling further genetic improvement and more effective use of this tree species.

Keywords: *Bombax ceiba*, genome assembly, annotation, evolution.

Data description

Introduction

Bombax ceiba Linn. (Malvaceae), commonly known as the cotton tree or red silk cotton tree, is a spectacular flowering tree with a height of up to 40 meters (Fig. 1a) that is found in tropical and sub-tropical Asia, and northern Australia [1]. It has been chosen as the “city flower” of the cities of Kaohsiung and Guangzhou in China for its large, showy flowers with thick, waxy, red petals that densely clothe leafless branch tips in late winter and early spring (Fig. 1b, c). *B. ceiba* is a source of food, fodder, fiber, fuel, medicine, and many other valuable goods for natives of many Asian countries [2]. For example, its fruits are good sources of silk-cotton for making mattresses, cushions, pillows and quilts [3], while its timbers are widely used in matches, boxes, and splints [4]. Moreover, studies on the cotton tree have shown that it produces many novel secondary metabolites and have explored its traditional medicinal usage by various tribal communities [1, 2, 5, 6]. In addition to its economic and medicinal value, *B. ceiba* is an ecologically important plant: it is a reforestation pioneer that survives easily in low-rainfall and well-drained conditions [7], and has been identified as a plant species suitable for municipal greening because of its capacity to counteract the detrimental effects of air pollution [8, 9].

Despite the considerable economic and ecological importance of *B. ceiba*, the genomic information available for this species is limited, which has hindered its utilization. Here we report a draft genome sequence for *B. ceiba* that is expected to facilitate and expand its use.

Sampling and sequencing

All samples were collected from Yuanmou, Yunnan Province, China (25°40′50.06″ N, 101°53′27.76″

E). Genomic DNA was extracted from leaves of a single tree using the Plant Genomic DNA kit (Tiangen, Beijing, China). A SMRTbell DNA library was then prepared and sequenced using P6, C4 chemistry according to the manufacturer's protocols (Pacific Biosciences), and a 20-kb SMRTbell library was generated using a BluePippin DNA size selection instrument (Sage Science) with a lower size limit of 10 kb. Single-molecule real-time sequencing of long reads was conducted on a PacBio Sequel platform with 19 SMRT cells. A total of 86.0 Gb of genomic data with an average read length of 8.4kb was generated after quality filtering (Table S1). In addition, a separate paired-end (PE) DNA library with an insert size of 400 bp (amplification by eight PCR cycles) was constructed and sequenced using the Illumina platform (PE 150) to enable a genome survey. The NGS sequencing produced 36.1 Gb of raw data, of which 20.0 Gb retained after filtering.

Total RNA was extracted from the bud, root, bark, flower, and fruit tissues of one *B. ceiba* individual using the QIAGEN RNeasy Plant Mini Kit (QIAGEN, Hilden, Germany). RNA-seq libraries were then prepared using the TruSeq RNA Library Preparation Kit (Illumina, CA, USA), and paired-end sequencing with a read length of 150 bp was conducted on the HiSeq 2000 platform, yielding 44.41 Gb of clean data (30,816,034—51,191,192 reads per sample) (Table S2).

Genome size and heterozygosity estimation

The genome size of *B. ceiba* was estimated by the K-mer method [10], using sequencing data from the Illumina DNA library. Quality-filtered reads were subjected to 17-mer frequency distribution analysis using the Jellyfish program [10]. The count distribution of 17-mers followed a Poisson distribution, with the highest peak occurring at a depth of 22 (Table S3 and Fig. S1). The estimated genome size was approximately 809,166,127 bp, and the heterozygosity rate of the *B. ceiba* genome was approximately

0.88%.

Genome assembly

Genome assembly was performed on full PacBio long reads using FALCON v0.3.0 (https://github.com/PacificBiosciences/falcon). Error correction and pre-assembly were carried out with the FALCON pipeline, after evaluating the outcomes of using different parameters in FALCON during the pre-assembly process. Based on the contig N50 results, a length_cutoff of 11kb and a length_cutoff_pr of 11.5kb for the assembly step were ultimately chosen. The draft assembly was polished using Arrow (https://github.com/PacificBiosciences/GenomicConsensus), which mapped the PacBio reads to the assembled genome with the Blasr pipline [11]. The preliminary genome assembly was approximately 852Mb in size, with a contig N50 size of 727Kb. A GC depth analysis was conducted to assess the potential contamination during sequencing and the coverage of the assembly, revealing that the genome had an average GC content of 33.3% and a unimodal GC content distribution (Fig. S2). The GC depth as well as the sequencing depth of the genome assembly suggested that there was no contamination from other species (Fig. S3). To further assess contaminations, we randomly selected some sequences from the genome assembly and searched the data against the NCBI database of bacteria with BLASTN [12] (E-value≤1e−5). No contamination from bacteria was detected.

Scaffolding with BioNano optical mapping

The purified genomic DNA of *B. ceiba* was embedded in an agarose layer and then labeled and counterstained using the protocol provided with the IrysPrep Reagent Kit (BioNano Genomics). Samples were then loaded into IrysChips and imaged on an Irys imaging instrument (BioNano

Genomics). After filtering using a molecule length cutoff of < 150Kb, a molecule SNR of < 2.75, a label SNR of < 2.75, and a label intensity of > 0.8, 160.0 Gb of BioNano clean data were obtained, with the N50 size of the labeled single molecules being 269.9 kb (Table S4).

A molecular quality report was generated by aligning the BioNano library sequences to the initial PacBio genome assembly, yielding a map rate of 34.2%. Using the PacBio genome assembly data as a reference, a reference genome assembly was conducted based on the clean BioNano data, yielding a consensus genome map of 1.09 Gb with an N50 of 0.7 Mb. To obtain a longer scaffold, the *de novo* assembly of PacBio reads was then mapped to the BioNano single-molecule genomic map. After scaffolding, the contig assembly contained 3,105 scaffolds with a scaffold N50 of 1.5Mb.

To fill the gaps in the scaffolds, the Blasr pipline [11] was used to map the PacBio long reads to the draft genome assembly scaffolding with BioNano optical mapping. The draft was polished using PBJelly 2 software [13] over three iterations. Reads from the Illumina DNA library (400bp) were then aligned against the genome assembly using the BWA software to fill the gaps and correct potential sequencing errors of the assembly, and a mapping rate of 99.2% was achieved [14]. The final assembly was polished using Pilon [15], yielding a final draft genome of approximately 895 Mb, with contig and scaffold N50 sizes of 1.0 Mb and 2.06 Mb, respectively (Table S5).

Evaluation of the completeness of the genome assembly gene space

To evaluate the coverage of the assembly, we aligned all the RNA-seq reads against the *B. ceiba* genome assembly using HISAT [16] with default parameters. The percentage of aligned reads ranged from 84.78% to 91.08% (Table S2). We then used Benchmarking Universal Single-Copy Orthologs (BUSCO) [17] to search the annotated genes in the assembly for the 1440 single-copy genes conserved

among all embryophytes. About 94.4% of the complete BUSCOs were found in the assembly (Table S6). These results suggested that the genome assembly was complete and robust.

Genome annotation

The repeat sequences in the genome consisted of simple sequence repeats (SSRs), moderately repetitive sequences, and highly repetitive sequences. The MISA tool [18] was used to search for SSR motifs in the *B. ceiba* genome, with default parameters. A total of 454,435 SSRs were identified in this way: 310,369, 105,004, 30,925, 6,448, 1,165 and 524 mono-, di-, tri-, tetra-, penta-, and hexa-nucleotide repeats, respectively (Table S7).

To identify known transposable elements (TEs) in the *B. ceiba* genome, RepeatMasker [19] was used to screen the assembled genome against the Repbase (v. 22.11) [20] and Mips-REdat libraries [21]. In addition, *de novo* evolved transposable element annotation was performed using RepeatModeler (v. 1.0.11) [19]. The combined results of the homology-based and *de novo* predictions indicated that repeated sequences account for 60.3% of the *B. ceiba* genome assembly (Table S8), with TEs comprising 60.30% of the repeated sequences, and long terminal repeats (LTRs) accounting for the greatest proportion (47.86%) of TEs (Table S8).

Homology-based ncRNA annotation was performed by mapping plant rRNA, miRNA and snRNA genes from the Rfam database (release 13.0) [22] to the *B. ceiba* genome using BLASTN [12] (E-value $\leq$1e−5). The tRNAscan-SE (v1.3.1) [23] program was used (with default parameters for eukaryotes) for tRNA annotation. RNAmmer v1.2 [24] was used to predict rRNAs and their subunits. These analyses identified 496 miRNAs, 894 tRNAs, 6,772 rRNAs, and 727 snRNAs (Table S9).

The homology-based and *de novo* predictions were also used to annotate protein coding genes. For

homology-based predictions, protein sequences from four species (*Arabidopsis thaliana*, *Carica papaya*, *Gossypium arboretum* and *Theobroma cacao*) (Table S10) were mapped onto the *B. ceiba* genome; the aligned sequences and the corresponding query proteins were then filtered and passed to GeneWise v2.4.1 [25] to search for accurately spliced alignments. For the *de novo* predictions, we first randomly selected 1000 full-length genes from the homology-based predictions to train model parameters for Augustus v3.0 [26], GeneID v1.4.4 [27], GlimmerHMM [28] and SNAP [29]. Augustus v3.0 [26], GeneID v1.4.4 [27], GlimmerHMM [28] and SNAP [29] were then used to predict genes based on the training set. Finally, EVidenceModeler (EVM) v1.1.1 [30] was used to integrate the predicted genes and generate a consensus gene set (Table S10). Genes with transposable elements were discarded using the TransposonPSI (http://transposonpsi.sourceforge.net/) package. Low-quality genes consisting of fewer than 50 amino acids and/or exhibiting premature termination were also removed from the gene set, yielding a final set of 52,705 genes. The final set's average transcript length, average CDS length and exon number per gene were 2,418.37 bp, 1,019.38 bp and 4.57, respectively (Table S11, Fig. S4).

The annotations of the predicted genes of *B. ceiba* were screened for homology against the Uniprot (release 2017_10) and KEGG (release 84.0) databases using Blastall [12] and KAAS [31]. Then, the InterProScan [32] package was used to annotate the predicted genes using the InterPro (5.21-60.0) database. In total, 47,105 of the total 52,705 genes (89.37%) were annotated with potential functions (Table S12).

Phylogenetic tree construction and divergence time estimation

To investigate the evolutionary position of *B. ceiba*, we compared its genome to the genome sequences

of 12 other plants. These included four plants in the Malvales order (*Gossypium arboreum*, *Durio zibethinus*, *Corchorus olitorius* and *Theobroma cacao*), seven plants from different orders in the same Eudicots clade (*Arabidopsis thaliana*, *Carica papaya*, *Linum usitatissimum*, *Populus trichocarpa*, *Camellia sinensis*, *Solanum lycopersicum* and *Vitis vinifera*), and *Oryza sativa* as an outgroup. Genome sequences from *A. thaliana*, *T. cacao*, *C. papaya*, *L. usitatissimum*, *P. trichocarpa*, *C. sinensis*, *S. lycopersicum*, *V. vinifera* and *O. sativa* were downloaded from Phytozome v. 12.0 [33]. Gene sequences of *G. arboreum*, *C. olitorius* and *D. zibethinus* were downloaded from the NCBI Database (PRJNA335838, PRJNA215141 and PRJNA400310). We used the OrthoMCL (v2.0.9) pipeline [34] (BLASTP E-value≤1e−5) to identify potentially orthologous gene families within these genomes. Gene family clustering identified 16,586 gene families containing 37,736 genes in *B. ceiba* (Fig. 2a). Of these, 906 gene families were unique to *B. ceiba* (Table S13). *B. ceiba* and other Malvales plants had the largest number of shared gene families among the studied plants.

Phylogenetic analysis was performed using 172 single copy orthologous genes from common gene families found by OrthoMCL [34] (Fig. S5). We codon-aligned each gene family using MUSCLE [35], and curated the alignments with Gblocks v0.91b [36]. Phylogeny analysis was performed using RAxML v 8.2.11[37] with the GTRGAMMA model and 100 bootstrap replicates. We then used MCMCTREE as implemented in PAML v4.9e [38] to estimate the divergence times of *B. ceiba* from the other plants. The parameter settings of MCMCTREE were as follows, clock=2, RootAge≤1.73, model=7, BDparas =110, kappa_gamma = 62, alpha_gamma = 11, rgene_gamma = 23.18, and sigma2_gamma = 14.5. In addition, the divergence times of *O. sativa* (148-173 Mya), *V. vinifera* (110-124Mya) and *A. thaliana* (53-82 Mya) were used for fossil calibration. The phylogenetic analysis showed that *B. ceiba* is more closely related to *G. arboraum* than to *D. zibethinus* (Fig. S6), which

supports the well-established hypothesis of a close relationship between Bombacaceae and Malvaceae [39, 40]. Recent phylogenetic studies have suggested that the group traditionally referred to as Bombacaceae (which includes the tribe Durioneae) is not actually monophyletic, and that the genera of the tribe Durioneae should be excluded from Bombacaceae. Most members of the erstwhile family Bombacaceae have been transferred to the subfamily Bombacoideae within the family Malvaceae [40]. This phylogenetic ordering was supported by our phylogenetic analysis of the complete chloroplast genomes of Marvel plants [41]. The estimated divergence time of *B. ceiba* and *D. zibethinus* was 29.5 million years ago, while that of *B. ceiba* and *G. arboretum* was about 20.6 million years ago (Fig. 2b).

Genes under positive selection

B. ceiba is an ecologically important plant which could survive in extreme climate conditions, such as hot-dry valley [7]. According to the neutral evolution theory of Darwin [42], the ratio of nonsynonymous substitution rate (Ka) and synonymous substitution rate (Ks) of protein coding genes could be used to identify genes under natural selection. So we calculated the average Ka/Ks values and conducted the branch-site likelihood ratio test using Codeml implemented in PAML package [38] to identify positively selected genes in the *B. ceiba* lineage. These genes might contribute to the adaption of unfavorable environments. Thirty-six positively selected genes were identified ($P \leq 0.05$). Of which, 32 genes could be annotated with potential functions in the Swissport database (Table S14). One gene was homolog to desiccation protectant protein coding gene (*Lea14*). There was a strong association of LEA proteins with abiotic stress tolerance particularly dehydration and cold stress [43]. This gene could potentially contribute to the adaption of *B. ceiba* to the dry valley environment. Another gene was homolog to the gene coding Kelch domain-containing protein 4. The Kelch domain-containing

proteins were involved in regulating a number of major processes such as growth, development, and biotic and abiotic stress responses in plants [44, 45]. The E3 ubiquitin-protein ligase (RFWD3) was suggested by some researchers that it had potential roles in plant stress responses [46, 47]. Twenty-one positively selected sites were identified in the CACTIN protein coding gene. The CACTIN protein was characterized as a negative regulator of many different developmental processes, such as embryogenesis [48]. While there were rare literature reports, other identified genes might also be associated with the ecological adaption of *B. ceiba*. It should be noted that this was just a primarily analysis of functions of these genes, further studies would be needed to clarify the roles of these genes.

Whole-genome duplication and Gene family expansion analysis

We used four-fold synonymous third-codon transversion (4DTv) estimation to detect whole-genome duplication (WGD) events in the *B. ceiba* genome. To this end, paralogous sequences of *B. ceiba*, *T. cacao*, *V. vinfira*, *S. lycopersicum* and *D. zibethinus* was identified with OrthoMCL [34]. Then, protein sequences for each of these plants were aligned against one-other with Blastp [12] (using an E-value threshold of ≤1e−5) to identify conserved paralogs in each species. Finally, potential WGD events in each genome were evaluated based on their 4DTv distribution. The WGD analysis suggested that *B. ceiba* experienced the same same WGD events as other Dicotyledons, and that *B. ceiba* and *D. zibethinus* went through their WGD events before diverging from their common ancestor (Fig. 2c).

The OrthoMCL gene family analysis results were analyzed further by using CAFE (Computational Analysis of gene Family Evolution, v3.0) [49] to detect expanded gene families. This approach revealed 5,612 expanded gene families and 1,902 contracted gene families in the *B. ceiba* lineage (Fig. S7).

Conclusion

This paper reports the sequencing, assembly, and annotation of the *B. ceiba* genome along with details of its evolutionary history. The genomic data generated in this work will be a valuable resource for further genetic improvement and effective use of the red silk cotton tree.

Availability of supporting data

The raw data from our genome project was deposited in the SRA (Sequence Read Archive) database of national center for biotechnology information with Bioproject ID PRJNA429932. The assembly and annotation of the *B. ceiba* genome are available in the GigaScience GigaDB database. Versions and main parameters of the software used in this study are provided in Table S15 in the supplementary file.

Competing interests

S. S. is an employee of Nextomics Bioscences. Other authors declare that they have no competing interests.

Authors' contributions

L. T. and B. T. designed the project; H. W., C. L. and H. C. collected samples and extracted the DNA and RNA samples; Y. G., S. S., H. W. and C. L. worked on sequencing and data analyzing; Y. G. wrote the manuscript; L. T., B. T. and D. D. revised the manuscript; All authors read and approved the final version of the manuscript.

Acknowledgements

We thank Guanglong Ou, Jianmei Wu and Renbin Zhu for offering photos of *B. ceiba*. This study was financially supported by the National Science Foundation of China (grant 31460561, 31760103,

31460179 and 31660680), the Key Laboratory of Forest Resources Conservation and Utilization in the Southwest Mountains of China (Southwest Forestry University), Ministry of Education, and the Yunnan Applied Basic Research Project (grant 2017FD145).

References

1. Barwick M. Tropical and Subtropical Trees. Portland, OR: Timber Press; 2004.
2. Jain V , Verma SK. Pharmacology of *Bombax Ceiba* Linn. Berlin Heidelberg: Springer; 2012.
3. Chand S , Singh AK. In Vitro Propagation of *Bombax Ceiba* L. (Silkcotton). Silvae Genetica. 1999;48 (6):313-7.
4. Nair GS , Bai Y. Ethnobotanical Value of Dry, Fallen Ovaries of *Bombax Ceiba* L. (Bombacaceae: Malvales). Journal of Threatened Taxa. 2012;4 (15):3443-6.
5. Ngwuluka NC. Are *Bombax Buonopozense* and *Bombax Malabaricum* Possible Nutraceuticals for Age Management? Preventive Medicine. 2012;54 (S3):64-70.
6. Pankaj HC , Somshekhar SK. *Bombax Ceiba* Linn.: Pharmacognosy, Ethnobotany and Phyto-Pharmacology. Pharmacognosy Communications. 2012;2 (3):2-9.
7. Zhou Z, Ma H, Lin K, et al. RNA-Seq Reveals Complicated Transcriptomic Responses to Drought Stress in a Nonmodel Tropic Plant, *Bombax Ceiba* L. Evolutionary Bioinformatics. 2015;11 (S1):27-37.
8. Peng C, Wen D, Sun Z, et al. Response of Some Plants for Municipal Greening to Air Pollutants. Journal of Tropical and Subtropical Botany. 2002;10 (4):321-7.
9. Elhagrassi AM, Ali MM, Osman AF, et al. Phytochemical Investigation and Biological Studies of *Bombax Malabaricum* Flowers. Natural Product Research. 2011;25 (2):141-51.
10. Marçais G , Kingsford C. A Fast, Lock-Free Approach for Efficient Parallel Counting of Occurrences of K-Mers. Bioinformatics. 2011;27 (6):764-70.
11. Chaisson MJ , Tesler G. Mapping Single Molecule Sequencing Reads Using Basic Local Alignment with Successive Refinement (Blasr): Application and Theory. BMC Bioinformatics. 2012;13 (1):238.
12. Camacho C, Coulouris G, Avagyan V, et al. Blast+: Architecture and Applications. BMC Bioinformatics. 2009;10 (1):421.
13. Worley KC, English AC, Richards S, et al. Improving Genomes Using Long Reads and Pbjelly 2. In: *International Plant and Animal Genome Conference Xxii* 2014.
14. Li H , Durbin R. Fast and Accurate Short Read Alignment with Burrows–Wheeler Transform. Oxford University Press; 2009.
15. Walker BJ, Abeel T, Shea T, et al. Pilon: An Integrated Tool for Comprehensive Microbial Variant Detection and Genome Assembly Improvement. Plos One. 2014;9 (11):e112963.
16. Kim D, Langmead B , Salzberg SL. Hisat: A Fast Spliced Aligner with Low Memory Requirements. Nature Methods. 2015;12 (4):357-60.
17. Simão FA, Waterhouse RM, Ioannidis P, et al. Busco: Assessing Genome Assembly and Annotation Completeness with Single-Copy Orthologs. Bioinformatics. 2015;31 (19):3210-2.
18. Thiel T, Michalek W, Varshney RK, et al. Exploiting Est Databases for the Development and Characterization of Gene-Derived Ssr-Markers in Barley (*Hordeum Vulgare* L.). Theoretical

and Applied Genetics. 2003;106 (3):411-22.

19. Tarailograovac M , Chen N. Using Repeatmasker to Identify Repetitive Elements in Genomic Sequences. 2009;3:4-14.

20. Bao W, Kojima KK , Kohany O. Repbase Update, a Database of Repetitive Elements in Eukaryotic Genomes. Mobile DNA. 2015;6 (1):11.

21. Thomas N, Martis MM, Roessner SK, et al. Mips Plantsdb: A Database Framework for Comparative Plant Genome Research. Nucleic Acids Research. 2013;41:1144-51.

22. Kalvari I, Argasinska J, Quinones-Olvera N, et al. Rfam 13.0: Shifting to a Genome-Centric Resource for Non-Coding RNA Families. Nucleic Acids Research. 2017; doi:https://doi.org/10.1093/nar/gkx1038.

23. Lowe TM , Eddy SR. Trnascan-Se: A Program for Improved Detection of Transfer RNA Genes in Genomic Sequence. Nucleic Acids Research. 1997;25 (5):955-64.

24. Lagesen K, Hallin P, Rødland EA, et al. RNAmmer: Consistent and Rapid Annotation of Ribosomal Rna Genes. Nucleic Acids Research. 2007;35 (9):3100-8.

25. Birney E , Durbin R. Using Genewise in the Drosophila Annotation Experiment. Genome Research. 2000;10 (4):547-8.

26. Stanke M, Steinkamp R, Waack S, et al. Augustus: A Web Server for Gene Finding in Eukaryotes. Nucleic Acids Research. 2004;32:309-12.

27. Blanco E, Parra G , Guigó R. Using Geneid to Identify Genes. Current protocols in bioinformatics. 2007; 4 (3):1-28.

28. Majoros WH, Pertea M , Salzberg SL. Tigrscan and Glimmerhmm: Two Open Source Ab Initio Eukaryotic Gene-Finders. Bioinformatics. 2004;20 (16):2878-9.

29. Bromberg Y , Rost B. Snap: Predict Effect of Non-Synonymous Polymorphisms on Function. Nucleic Acids Research. 2007;35 (11):3823-35.

30. Haas BJ, Salzberg SL, Wei Z, et al. Automated Eukaryotic Gene Structure Annotation Using Evidencemodeler and the Program to Assemble Spliced Alignments. Genome Biology. 2008;9 (1):R7.

31. Moriya Y, Itoh M, Okuda S, et al. Kaas: An Automatic Genome Annotation and Pathway Reconstruction Server. Nucleic Acids Research. 2007;35:W182-W5.

32. Quevillon E, Silventoinen V, Pillai S, et al. Interproscan: Protein Domains Identifier. Nucleic Acids Research. 2005;33:116-20.

33. M GD, Shengqiang S, Russell H, et al. Phytozome: A Comparative Platform for Green Plant Genomics. Nucleic acids research. 2012;40 (Database issue):D1178-D86.

34. Li L, Stoeckert CJ , Roos DS. Orthomcl: Identification of Ortholog Groups for Eukaryotic Genomes. Genome Research. 2003;13 (9):2178-89.

35. Edgar RC. Muscle: Multiple Sequence Alignment with High Accuracy and High Throughput. Nucleic Acids Research. 2004;32 (5):1792-7.

36. Talavera G , Castresana J. Improvement of Phylogenies after Removing Divergent and Ambiguously Aligned Blocks from Protein Sequence Alignments. Systematic Biology. 2007;56 (4):564-77.

37. Stamatakis A. Raxml Version 8: A Tool for Phylogenetic Analysis and Post-Analysis of Large Phylogenies. Bioinformatics. 2014;30 (9):1312-3.

38. Yang Z. Paml 4: Phylogenetic Analysis by Maximum Likelihood. Molecular Biology and Evolution. 2007;24 (8):1586-91.

39. Baum DA, Smith DW, Yen A, et al. Phylogenetic Relationships of Malvatheca (Bombacoideae and Malvoideae; Malvaceae Sensu Lato) as Inferred from Plastid DNA Sequences. American Journal of Botany. 2004;91 (11):1863-71.
40. Heywood, V.H, Brummitt, et al. Flowering Plant Families of the World. Richmond, Surrey: Royal Botanic Gardens; 2007.
41. Gao Y, Wang H, Liu C, et al. Complete Chloroplast Genome Sequence of the Red Silk Cotton Tree (*Bombax Ceiba*). Mitochondrial DNA Part B. 2018;3 (1):315-6. doi:10.1080/23802359.2017.1422399.
42. Darwin C. On the Origin of Species by Means of Natural Selectionor, Thepreservation of Favoured Races in the Struggle for Life. American Anthropologist. 1951;61 (1):176-7.
43. Shinde S, Nurul IM , Ng CK. Dehydration Stress-Induced Oscillations in LEA Protein Transcripts Involves Abscisic Acid in the Moss, *Physcomitrella Patens*. New Phytologist. 2012;195 (2):321-8.
44. Feder A, Burger J, Gao S, et al. Focus on Metabolism: A Kelch Domain-Containing F-Box Coding Gene Negatively Regulates Flavonoid Accumulation in Muskmelon. Plant Physiology. 2015;169 (3):1714-26.
45. Zhang X, Gou M, Guo C, et al. Down-Regulation of Kelch Domain-Containing F-Box Protein in Arabidopsis Enhances the Production of (Poly)Phenols and Tolerance to Ultraviolet Radiation. Plant Physiology. 2015;167 (2):337-50.
46. Serrano I, Campos L , Rivas S. Roles of E3 Ubiquitin-Ligases in Nuclear Protein Homeostasis During Plant Stress Responses. Frontiers in Plant Science. 2018;9 (139) doi:10.3389/fpls.2018.00139.
47. Duplan V , Rivas S. E3 Ubiquitin-Ligases and Their Target Proteins During the Regulation of Plant Innate Immunity. Frontiers in Plant Science. 2014;5 (42) doi:10.3389/fpls.2014.00042.
48. Baldwin KL, Dinh EM, Hart BM, et al. CACTIN is an Essential Nuclear Protein in *Arabidopsis* and may be Associated with the Eukaryotic Spliceosome. Febs Letters. 2013;587 (7):873-9.
49. De Bie T, Cristianini N, Demuth JP, et al. Cafe: A Computational Tool for the Study of Gene Family Evolution. Bioinformatics. 2006;22 (10):1269-71.

Figure 1. Example of the red silk cotton tree (*B. ceiba*). (a) Natural habitat of *B. ceiba* (image from Guanglong Ou). (b) *B. ceiba* used as municipal greening trees (image from Jianmei Wu). (c) The flower of *B. ceiba* (image from Renbin Zhu).

Figure 2. Phylogenetic relationships and genomic comparisons between *B. ceiba* and other plants. (a) A Venn diagram of shared gene families between *B. ceiba* and three other Malvales plants, with *A. thaliana* as an outgroup. Each number represents a gene family number. (b) Inferred phylogenetic tree

across 13 plant species. The estimated divergence time (Mya) is shown at each node. (c) WGD events of four plants (*B. ceiba*, *D. zibethinus*, *S. lycopersicum* and *V. vinifera*) inferred by 4DTv estimations. Peaks corresponding to speciation, recent and ancient WGDs are indicated by arrows.

Additional files

Figure S1. Frequency distribution of the 17-mer graph analysis used to estimate the size of the *B. ceiba* genome.

Figure S2. GC content distribution of the *B. ceiba* genome. The GC content was established using 500 bp sliding windows.

Figure S3. The GC depth distribution of the *B. ceiba* genome.

Figure S4. Comparison of gene structure characteristics in *B. ceiba* to that in other plants. a, CDS length; b, Exon length; c, Exon number; d, Gene length; e, Intron length.

Figure S5. Gene orthology determined by comparing genomes using the OrthoMCL software.

Figure S6. The maximum-likelihood phylogeny of *B. ceiba* and 13 other plants.

Figure S7. Gene family expansions and contractions in *B. ceiba* and 13 other plants.

Table S1. Sequencing statistics from the PacBio platform

Table S2. Summary of the transcriptomes and their mapping rates on the genome assembly

Table S3. Estimation of genome size based on 17-mer statistics

Table S4. Summary of the BioNano optical mapping data

Table S5. Summary of the final genome assembly

Table S6. Summary of BUSCO analysis results

Table S7. Summary of the SSR search results

Table S8. Repeat annotation of the *Bombax ceiba* genome assembly

Table S9. Summary of non-protein-coding gene annotations in the *Bombax ceiba* genome assembly

Table S10. Gene annotation statistics of the *Bombax ceiba* genome assembly

Table S11. Comparative gene statistics

Table S12. Functional annotation of predicted genes of *Bombax ceiba*

Table S13. Summary statistics of gene families in 13 plant species

Table S14. Candidate positively selected genes in the *Bombax ceiba* lineage

Table S15. Versions and main parameters of the software used in this study

Figure 1

Click here to download Figure renamed_51f32.tif

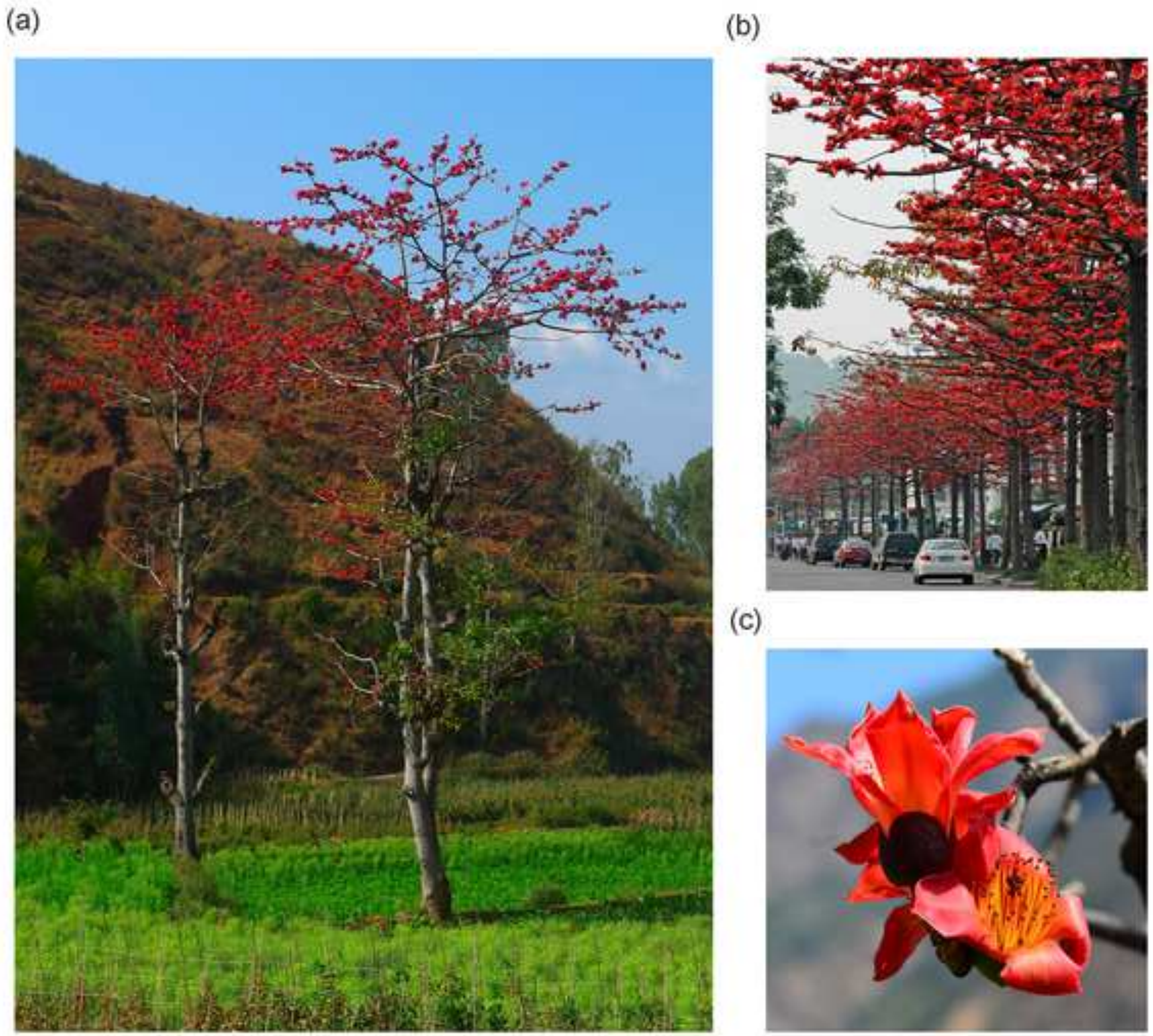

Figure 2

Click here to download Figure Figure 2.eps

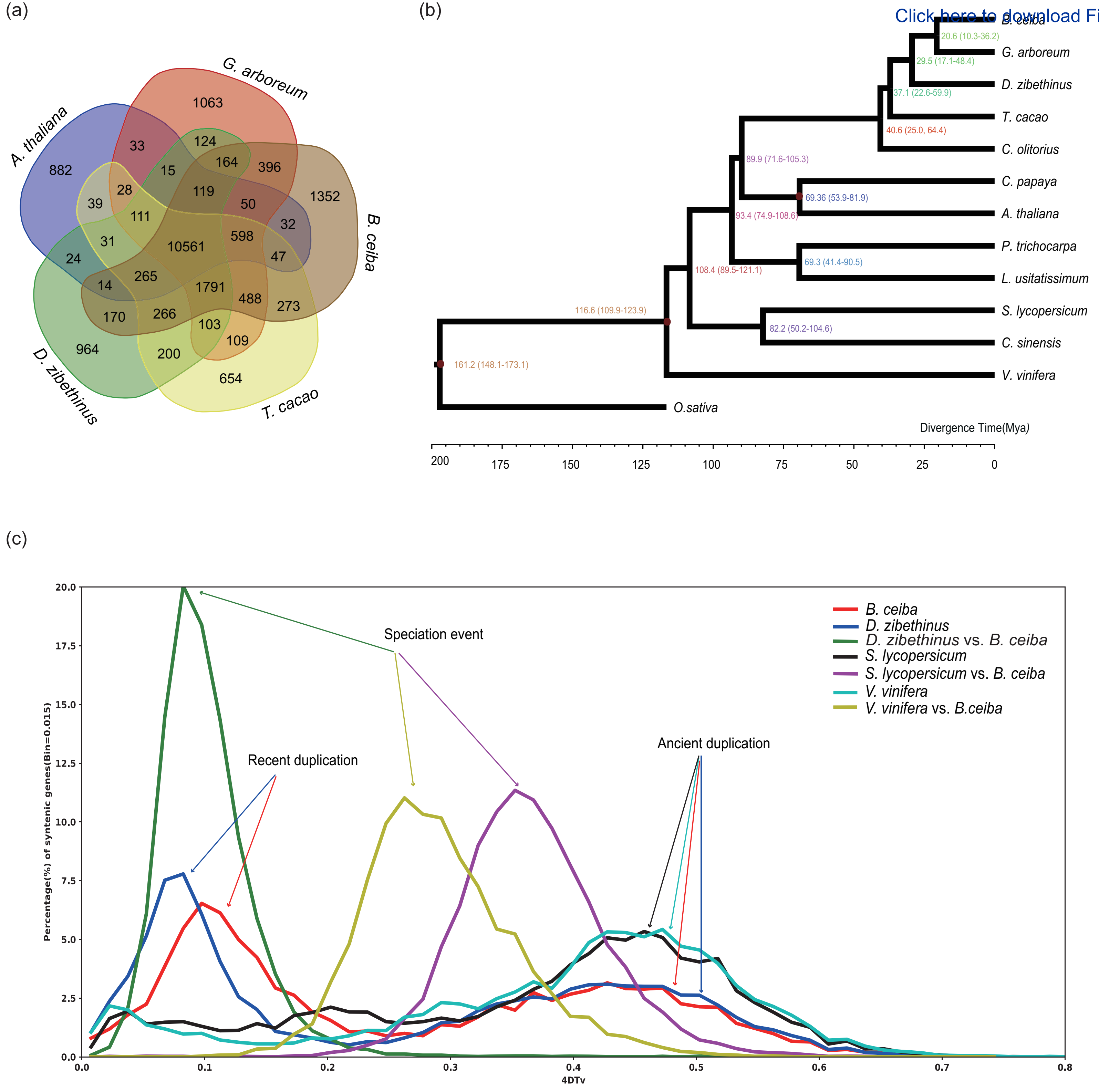

Click here to access/download
Supplementary Material
Supplementary file20180309.docx
