# Supplementary material for: De novo genome assembly of the red silk cotton tree (Bombax ceiba)
Source: Gigascience. 2018 May 10;7(5):giy051. doi: 10.1093/gigascience/giy051 (PMC5967522; doi:10.1093/gigascience/giy051)
Supplement: GIGA-D-18-00045_Revision_2.pdf [file giy051_giga-d-18-00045_revision_2.pdf]

GigaScience

De novo genome assembly of the red silk cotton tree (Bombax ceiba)

--Manuscript Draft--

**Manuscript Number:**	GIGA-D-18-00045R2
**Full Title:**	De novo genome assembly of the red silk cotton tree (Bombax ceiba)
**Article Type:**	Data Note
**Funding Information:**	National Natural Science Foundation of China (31460561) — Dr. Lizhou Tang<br>National Natural Science Foundation of China (31760103) — Dr. Yong Gao<br>National Natural Science Foundation of China (31460179) — Dr. Haibo Wang<br>National Natural Science Foundation of China (31660680) — Dr. Long Yu<br>Applied Basic Research Key Project of Yunnan (2017FD145) — Dr. Yong Gao
**Abstract:**	Background: Bombax ceiba L. (the red silk cotton tree) is a large deciduous tree that is distributed in tropical and sub-tropical Asia, and northern Australia. It has great economic and ecological importance, with several applications in industry and traditional medicine in many Asian countries. To facilitate the further utilization of this plant resource, we present here the draft genome sequence for B. ceiba.<br>Findings: We assembled a relatively intact genome of B. ceiba by using PacBio single-molecule sequencing and BioNano optical mapping technologies. The final draft genome is approximately 895 Mb long, with contig and scaffold N50 sizes of 1.0 Mb and 2.06 Mb, respectively.<br>Conclusions: The high-quality draft genome assembly of B. ceiba will be a valuable resource enabling further genetic improvement and more effective use of this tree species.
**Corresponding Author:**	Lizhou Tang<br>Center for Yunnan Plateau Biological Resources Protection and Utilization<br>Qujing, Yunnan CHINA
**Corresponding Author Secondary Information:**	
**Corresponding Author's Institution:**	Center for Yunnan Plateau Biological Resources Protection and Utilization
**Corresponding Author's Secondary Institution:**	
**First Author:**	Yong Gao
**First Author Secondary Information:**	
**Order of Authors:**	Yong Gao
	Haibo Wang
	Chao Liu
	Honglong Chu
	Dongqin Dai
	Shengnan Song
	Long Yu
	Lihong Han

Powered by Editorial Manager® and ProduXion Manager® from Aries Systems Corporation

	Yi Fu
	Bin Tian
	Lizhou Tang
**Order of Authors Secondary Information:**	
**Response to Reviewers:**	Dear editor and reviewers,<br>The manuscript "De novo genome assembly of the red silk cotton tree (Bombax ceiba)" (GIGA-D-18-00045R1) has been carefully revised according to the reviewers' suggestion. The major revisions are marked in red.<br><br>Reviewer #2:<br>1. For my previous question 1: the authors pointed out that heterozygosity may affect the estimation of genome size by using Kmers. Do authors believe <1% heterozygosity rate can lead to ~100 Mb assembly differences (the final assembly is 895Mb)? Is that possible 17-mer underestimated the genome size (I understand that in BGI's paper they used 17-mer to estimate giant panda's genome size. Is 17-mer suitable for B.ceiba? If authors test different kmers, I suppose you will have different results).<br>Answer: As the reviewer suggested, we reanalyzed the genome size with other K-mers (19-mer and 21-mer). The estimated genome size was 835 Mb (19-mer) and 821 Mb (21-mer), respectively. The results did not dramatically depart from the genome size estimated with 17-mer (809 Mb). And our previously study of flow cytometry also suggested that the genome size of B. ceiba was approximately 800Mb (2C =1.55±0.03pg) [1]. The heterozygosity rate of B. ceiba genome was 0.88%. As pointed by many researchers, genomes with heterozygosity rate higher than 0.5% are considered as highly heterozygous [2]. Assembling highly heterozygous diploid genomes is a substantial challenge, and heterozygous regions could not be assembled into consensus may result in larger assembly [2]. So we concluded that the highly heterozygous genome of B. ceiba might be the main reason why there were ~100 Mb differences between the estimated genome size and the final assembly.<br><br>2. For my previous question 2: I appreciate that authors used BLASTN to confirm contaminations. However, shouldn't authors use the non-plant database instead of bacteria? Why did authors randomly select some contigs (how many?) instead of all of them? I understand that using random selection to avoid bias, but since the contamination rate is low (I suppose), you will have less chance to select a contamination contig if you only select a few contigs from the pool.<br>Answer: We accepted the reviewer's suggestion, and we searched all sequences of the genome assembly against the NCBI nucleotide collection (Nt) with BLASTN with E-value < 1e-5 and sequence identity > 70%. In total, 2494 significant hits were achieved. The top-hit species were Theobroma cacao and Gossypium species, comprising more than 69% of the hits (1733 hits). Only five hits from four non-plant species (Psyllidae sp., Trioza eugeniae, Diptacus sp., and Dichorragia nesimachus) were detected, which suggested there was no potential contamination from non-plant species in the genome of Bombax ceiba.<br>Please see line 89-95 in the revised manuscript and Table S4 in the supplementary file.<br><br>3. For my previous question 3: I understand that there are some discrepancies between Bionano consensus maps and the NGS reference. I want to know how many Bionano consensus maps can align to the Pacbio assembly or the total size of the aligned PacBio assembly? Since Bionano gives 1.09Gb consensus maps, does that mean the real genome size of B.ceiba is around 1.09 Gb? Does that mean the Pacbio assembly is still underestimated? For the 'Ns' added through Bionano scaffolding, it can be easily checked.<br>Answer: Approximately 64.3 Gb (2023 individual maps) out of 160 Gb BioNano clean data could be mapped to the Pacbio assembly, and the average molecule coverage depth of the genome map was 27 × (Please check line 106-108 in the revised manuscript). The final genome assembly scaffolded by BioNano mapping contained 386 gaps (25,395,219 bp in total). The larger genome consensus map (1.09 Gb) should mainly be contributed to redundancies because of the high genome heterozygosity.<br><br>New questions:

	1. why did authors change the final assembled genome size from 869Mb to 895Mb, but didn't change any stats, is anything wrong with the previous calculation?<br>Answer: We are sorry for this matter. During the initial submission, we made a mistake and took the contig size (869Mb) as the assembled genome size due to negligence. So we changed the size of the final genome assembly to 895Mb (the scaffold size) in the revised manuscript. We appreciate very much for this comment.<br>2. From the density of Bionano label, it seems it is low. Which enzyme did authors use to generate the Bionano data? Is that Nt. BspQI?<br><br>Answer: We used Nt. BspQI enzyme in the BioNano mapping procedure. This information has been added to the revised manuscript. Please see line 98-99.<br><br>Finally, we appreciate the quick, detailed, useful comments and suggestions from the reviewers for improving our manuscript.<br><br>Reference<br><br>1.Zhou ZL, Xiong Z, Huan-Cheng MA, et al. Genome Sizes of Two Bombacaceae Plants. Journal of West China Forestry Science. 2014;43 (2):97-101. (in Chinese)<br>2.Kajitani R, Toshimoto K, Noguchi H, et al. Efficient De Novo Assembly of Highly Heterozygous Genomes from Whole-Genome Shotgun Short Reads. Genome Research. 2014;24 (8):1384-95.
**Additional Information:**	
**Question**	**Response**
Are you submitting this manuscript to a special series or article collection?	No
**Experimental design and statistics**<br><br>Full details of the experimental design and statistical methods used should be given in the Methods section, as detailed in our Minimum Standards Reporting Checklist. Information essential to interpreting the data presented should be made available in the figure legends.<br><br>Have you included all the information requested in your manuscript?	Yes
**Resources**<br><br>A description of all resources used, including antibodies, cell lines, animals and software tools, with enough information to allow them to be uniquely identified, should be included in the Methods section. Authors are strongly encouraged to cite Research Resource Identifiers (RRIDs) for antibodies, model organisms and tools, where possible.<br><br>Have you included the information requested as detailed in our Minimum Standards Reporting Checklist?	Yes

Powered by Editorial Manager® and ProduXion Manager® from Aries Systems Corporation

**Availability of data and materials**<br><br>All datasets and code on which the conclusions of the paper rely must be either included in your submission or deposited in publicly available repositories (where available and ethically appropriate), referencing such data using a unique identifier in the references and in the “Availability of Data and Materials” section of your manuscript.<br><br>Have you have met the above requirement as detailed in our Minimum Standards Reporting Checklist?	Yes

Powered by Editorial Manager® and ProduXion Manager® from Aries Systems Corporation

Manuscript

Click here to download Manuscript RedSilkCottonTree_GS_2_5_18.docx

De novo genome assembly of the red silk cotton tree (*Bombax ceiba*)

Yong Gao[1, #], Haibo Wang[1, #], Chao Liu[1, #], Honglong Chu[1], Dongqin Dai[1], Shengnan Song[5], Long Yu[1], Lihong Han[1], Yi Fu[2], Bin Tian[2,3, *], Lizhou Tang[1,4, *]

[1] Center for Yunnan Plateau Biological Resources Protection and Utilization, College of Biological Resource and Food Engineering, Qujing Normal University, Qujing, Yunnan, 655011, China

[2] Key Laboratory of Biodiversity Conservation in Southwest China, State Forestry Administration, Southwest Forestry University, Kunming 650224, China

[3] Key Laboratory of Biodiversity and Biogeography, Kunming Institute of Botany, Chinese Academy of Sciences, Kunming 650204, China

[4] State Key Laboratory of Genetic Resources and Evolution，Kunming Institute of Zoology，Chinese Academy of Sciences, Kunming 650223, China

[5] Nextomics Biosciences Institute, Wuhan, Hubei 430000, China

[#] These authors contributed equally to this work.

[*] Correspondence should be addressed to Lizhou Tang (tanglizhou@163.com) and Bin Tian (tianbinlzu@163.com).

***De novo* genome assembly of the red silk cotton tree (*Bombax ceiba*)**

Abstract

Background: *Bombax ceiba* L. (the red silk cotton tree) is a large deciduous tree that is distributed in tropical and sub-tropical Asia, and northern Australia. It has great economic and ecological importance, with several applications in industry and traditional medicine in many Asian countries. To facilitate the further utilization of this plant resource, we present here the draft genome sequence for *B. ceiba*.

Findings: We assembled a relatively intact genome of *B. ceiba* by using PacBio single-molecule sequencing and BioNano optical mapping technologies. The final draft genome is approximately 895 Mb long, with contig and scaffold N50 sizes of 1.0 Mb and 2.06 Mb, respectively.

Conclusions: The high-quality draft genome assembly of *B. ceiba* will be a valuable resource enabling further genetic improvement and more effective use of this tree species.

Keywords: *Bombax ceiba*, genome assembly, annotation, evolution.

Data description

Introduction

Bombax ceiba Linn. (Malvaceae), commonly known as the cotton tree or red silk cotton tree, is a spectacular flowering tree with a height of up to 40 meters (Fig. 1a) that is found in tropical and sub-tropical Asia, and northern Australia [1]. It has been chosen as the "city flower" of the cities of Kaohsiung and Guangzhou for its large, showy flowers with thick, waxy, red petals that densely clothe leafless branch tips in late winter and early spring (Fig. 1b, c). *B. ceiba* is a source of food, fodder, fiber, fuel, medicine, and many other valuable goods for natives of many Asian countries [2]. For example, its fruits are good sources of silk-cotton for making mattresses, cushions, pillows and quilts [3], while its timbers are widely used in matches, boxes, and splints [4]. Moreover, studies on the cotton tree have shown that it produces many novel secondary metabolites and have explored its traditional medicinal usage by various tribal communities [1, 2, 5, 6]. In addition to its economic and medicinal value, *B. ceiba* is an ecologically important plant: it is a reforestation pioneer that survives easily in low-rainfall and well-drained conditions [7], and has been identified as a plant species suitable for municipal greening because of its capacity to counteract the detrimental effects of air pollution [8, 9].

Despite the considerable economic and ecological importance of *B. ceiba*, the genomic information available for this species is limited, which has hindered its utilization. Here we report a draft genome sequence for *B. ceiba* that is expected to facilitate and expand its use.

Sampling and sequencing

All samples were collected from Yuanmou, Yunnan Province, China (25°40′50.06″ N, 101°53′27.76″ E). Genomic DNA was extracted from leaves of a single tree using the Plant Genomic DNA kit

(Tiangen, Beijing, China). A SMRTbell DNA library was then prepared and sequenced using P6, C4 chemistry according to the manufacturer's protocols (Pacific Biosciences), and a 20-kb SMRTbell library was generated using a BluePippin DNA size selection instrument (Sage Science) with a lower size limit of 10 kb. Single-molecule real-time sequencing of long reads was conducted on a PacBio Sequel platform with 19 SMRT cells. A total of 86.0 Gb of genomic data with an average read length of 8.4kb was generated after quality filtering (Table S1). In addition, a separate paired-end (PE) DNA library with an insert size of 400 bp (amplification by eight PCR cycles) was constructed and sequenced using the Illumina platform (PE 150) to enable a genome survey. The NGS sequencing produced 36.1 Gb of raw data, of which 20.0 Gb retained after filtering.

Total RNA was extracted from the bud, root, bark, flower, and fruit tissues of one *B. ceiba* individual using the QIAGEN RNeasy Plant Mini Kit (QIAGEN, Hilden, Germany). RNA-seq libraries were then prepared using the TruSeq RNA Library Preparation Kit (Illumina, CA, USA), and paired-end sequencing with a read length of 150 bp was conducted on the HiSeq 2000 platform, yielding 44.41 Gb of clean data (30,816,034—51,191,192 reads per sample) (Table S2).

Genome size and heterozygosity estimation

The genome size of *B. ceiba* was estimated by the K-mer method [10], using sequencing data from the Illumina DNA library. Quality-filtered reads were subjected to 17-mer frequency distribution analysis using the Jellyfish program [10]. The count distribution of 17-mers followed a Poisson distribution, with the highest peak occurring at a depth of 22 (Table S3 and Fig. S1). The estimated genome size was approximately 809,166,127 bp, and the heterozygosity rate of the *B. ceiba* genome was approximately 0.88%.

Genome assembly

Genome assembly was performed on full PacBio long reads using FALCON v0.3.0 (https://github.com/PacificBiosciences/falcon). Error correction and pre-assembly were carried out with the FALCON pipeline, after evaluating the outcomes of using different parameters in FALCON during the pre-assembly process. Based on the contig N50 results, a length_cutoff of 11kb and a length_cutoff_pr of 11.5kb for the assembly step were ultimately chosen. The draft assembly was polished using Arrow (https://github.com/PacificBiosciences/GenomicConsensus), which mapped the PacBio reads to the assembled genome with the Blasr pipline [11]. The preliminary genome assembly was approximately 852Mb in size, with a contig N50 size of 727Kb. A GC depth analysis was conducted to assess the potential contamination during sequencing and the coverage of the assembly, revealing that the genome had an average GC content of 33.3% and a unimodal GC content distribution (Fig. S2). The GC depth as well as the sequencing depth of the genome assembly suggested that there was no contamination from other species (Fig. S3). To further assess contaminations, we searched all sequences of the genome assembly against the NCBI non-redundant nucleotide database (Nt) with BLASTN [12] (E-value≤1e−5). In total, 2494 significant hits were achieved. The top-hit species were *Theobroma cacao* and *Gossypium* species, comprising more than 69% (1733 hits) of the hits (Table S4). Only five hits from four non-plant species (*Psyllidae* sp., *Trioza eugeniae*, *Diptacus* sp. and *Dichorragia nesimachus*) were detected (Table S4), suggesting there was no potential contamination from non-plant species in the genome of *Bombax ceiba*.

Scaffolding with BioNano optical mapping

The purified genomic DNA of *B. ceiba* was embedded in an agarose layer, digested with Nt. BspQI enzyme and labeled. The molecules were counterstained using the protocol provided with the IrysPrep Reagent Kit (BioNano Genomics). Samples were then loaded into IrysChips and imaged on an Irys imaging instrument (BioNano Genomics). After filtering using a molecule length cutoff of < 150Kb, a molecule SNR of < 2.75, a label SNR of < 2.75, and a label intensity of > 0.8, 160.0 Gb of BioNano clean data were obtained, with the N50 size of the labeled single molecules being 269.9 kb (Table S5).

A molecular quality report was generated by aligning the BioNano library sequences to the initial PacBio genome assembly, yielding a map rate of 34.2%. Using the PacBio genome assembly data as a reference, a reference genome assembly was conducted based on the clean BioNano data. A genome map consisting of 2023 consensus maps was assembled, yielding a genome size of 1.09 Gb with an N50 size of 0.7 Mb. The average molecule coverage depth of the genome map was about 27 folds. To obtain a longer scaffold, the *de novo* assembly of PacBio reads was then mapped to the BioNano single-molecule genomic map. After scaffolding, the contig assembly contained 3,105 scaffolds with a scaffold N50 of 1.5Mb.

To fill the gaps in the scaffolds, the Blasr pipline [11] was used to map the PacBio long reads to the draft genome assembly scaffolding with BioNano optical mapping. The draft was polished using PBJelly 2 (PBJelly, RRID:SCR_012091) [13] over three iterations. Reads from the Illumina DNA library (400bp) were then aligned against the genome assembly using the BWA software (BWA, RRID:SCR_010910) to fill the gaps and correct potential sequencing errors of the assembly, and a mapping rate of 99.2% was achieved [14]. The final assembly was polished using Pilon [15], yielding a final draft genome of approximately 895 Mb, with contig and scaffold N50 sizes of 1.0 Mb and 2.06 Mb, respectively (Table S6).

Evaluation of the completeness of the genome assembly gene space

To evaluate the coverage of the assembly, we aligned all the RNA-seq reads against the *B. ceiba* genome assembly using HISAT [16] with default parameters. The percentage of aligned reads ranged from 84.78% to 91.08% (Table S2). We then used Benchmarking Universal Single-Copy Orthologs (BUSCO, RRID:SCR_015008) [17] to search the annotated genes in the assembly for the 1440 single-copy genes conserved among all embryophytes. About 94.4% of the complete BUSCOs were found in the assembly (Table S7). These results suggested that the genome assembly was complete and robust.

Genome annotation

The repeat sequences in the genome consisted of simple sequence repeats (SSRs), moderately repetitive sequences, and highly repetitive sequences. The MISA tool [18] was used to search for SSR motifs in the *B. ceiba* genome, with default parameters. A total of 454,435 SSRs were identified in this way: 310,369, 105,004, 30,925, 6,448, 1,165 and 524 mono-, di-, tri-, tetra-, penta-, and hexa-nucleotide repeats, respectively (Table S8).

To identify known transposable elements (TEs) in the *B. ceiba* genome, RepeatMasker (RepeatMasker, RRID:SCR_012954) [19] was used to screen the assembled genome against the Repbase (v. 22.11) [20] and Mips-REdat libraries [21]. In addition, *de novo* evolved transposable element annotation was performed using RepeatModeler v. 1.0.11 (RepeatModeler, RRID:SCR_015027) [19]. The combined results of the homology-based and *de novo* predictions indicated that repeated sequences account for 60.30% of the *B. ceiba* genome assembly (Table S9),

with long terminal repeats (LTRs) accounting for the greatest proportion (47.86%) (Table S9).

Homology-based ncRNA annotation was performed by mapping plant rRNA, miRNA and snRNA genes from the Rfam database (release 13.0) [22] to the *B. ceiba* genome using BLASTN [12] (E-value ≤1e−5). tRNAscan-SE v1.3.1 (tRNAscan-SE, RRID:SCR_010835) [23] was used (with default parameters for eukaryotes) for tRNA annotation. RNAmmer v1.2 [24] was used to predict rRNAs and their subunits. These analyses identified 496 miRNAs, 894 tRNAs, 6,772 rRNAs, and 727 snRNAs (Table S10).

The homology-based and *de novo* predictions were also used to annotate protein coding genes. For homology-based predictions, protein sequences from four species (*Arabidopsis thaliana*, *Carica papaya*, *Gossypium arboretum* and *T. cacao*) (Table S11) were mapped onto the *B. ceiba* genome; the aligned sequences and the corresponding query proteins were then filtered and passed to GeneWise v2.4.1 (GeneWise, RRID:SCR_015054) [25] to search for accurately spliced alignments. For the *de novo* predictions, we first randomly selected 1000 full-length genes from the homology-based predictions to train model parameters for Augustus v3.0 (Augustus: Gene Prediction, RRID:SCR_008417) [26], GeneID v1.4.4 [27], GlimmerHMM (GlimmerHMM, RRID:SCR_002654) [28] and SNAP [29]. Augustus v3.0 [26], GeneID v1.4.4 [27], GlimmerHMM [28] and SNAP [29] were then used to predict genes based on the training set. Finally, EVidenceModeler (EVM) v1.1.1 [30] was used to integrate the predicted genes and generate a consensus gene set (Table S11). Genes with transposable elements were discarded using the TransposonPSI (http://transposonpsi.sourceforge.net/) package. Low-quality genes consisting of fewer than 50 amino acids and/or exhibiting premature termination were also removed from the gene set, yielding a final set of 52,705 genes. The final set's average transcript length, average CDS length and exon number per gene were 2,418.37 bp, 1,019.38

bp and 4.57, respectively (Table S12, Fig. S4).

The annotations of the predicted genes of *B. ceiba* were screened for homology against the Uniprot (release 2017_10) and KEGG (release 84.0) databases using Blastall [12] and KAAS [31]. Then, the InterProScan (InterProScan, RRID:SCR_005829) [32] package was used to annotate the predicted genes using the InterPro (5.21-60.0) database. In total, 47,105 of the total 52,705 genes (89.37%) were annotated with potential functions (Table S13).

Phylogenetic tree construction and divergence time estimation

To investigate the evolutionary position of *B. ceiba*, we compared its genome to the genome sequences of 12 other plants. These included four plants in the Malvales order (*Gossypium arboreum*, *Durio zibethinus*, *Corchorus olitorius* and *T. cacao*), seven plants from different orders in the same Eudicots clade (*Arabidopsis thaliana*, *Carica papaya*, *Linum usitatissimum*, *Populus trichocarpa*, *Camellia sinensis*, *Solanum lycopersicum* and *Vitis vinifera*), and *Oryza sativa* as an outgroup. Genome sequences from *A. thaliana*, *T. cacao*, *C. papaya*, *L. usitatissimum*, *P. trichocarpa*, *C. sinensis*, *S. lycopersicum*, *V. vinifera* and *O. sativa* were downloaded from Phytozome v. 12.0 [33]. Gene sequences of *G. arboreum*, *C. olitorius* and *D. zibethinus* were downloaded from the NCBI Database (PRJNA335838, PRJNA215141 and PRJNA400310). We used the OrthoMCL (v2.0.9) pipeline (OrthoMCL DB: Ortholog Groups of Protein Sequences, RRID:SCR_007839) [34] (BLASTP E-value≤1e−5) to identify potentially orthologous gene families within these genomes. Gene family clustering identified 16,586 gene families containing 37,736 genes in *B. ceiba* (Fig. 2a). Of these, 906 gene families were unique to *B. ceiba* (Table S14). *B. ceiba* and other Malvales plants had the largest number of shared gene families among the studied plants.

Phylogenetic analysis was performed using 172 single copy orthologous genes from common gene families found by OrthoMCL [34] (Fig. S5). We codon-aligned each gene family using MUSCLE (MUSCLE, RRID:SCR_011812) [35], and curated the alignments with Gblocks v0.91b [36]. Phylogeny analysis was performed using RAxML (RAxML, RRID:SCR_006086) v 8.2.11[37] with the GTRGAMMA model and 100 bootstrap replicates. We then used MCMCTREE as implemented in PAML v4.9e (PAML, RRID:SCR_014932) [38] to estimate the divergence times of *B. ceiba* from the other plants. The parameter settings of MCMCTREE were as follows, clock=2, RootAge≤1.73, model=7, BDparas =110, kappa_gamma = 62, alpha_gamma = 11, rgene_gamma = 23.18, and sigma2_gamma = 14.5. In addition, the divergence times of *O. sativa* (148-173 Mya), *V. vinifera* (110-124Mya) and *A. thaliana* (53-82 Mya) were used for fossil calibration. The phylogenetic analysis showed that *B. ceiba* is more closely related to *G. arboraum* than to *D. zibethinus* (Fig. S6), which supports the well-established hypothesis of a close relationship between Bombacaceae and Malvaceae [39, 40]. Recent phylogenetic studies have suggested that the group traditionally referred to as Bombacaceae (which includes the tribe Durioneae) is not actually monophyletic, and that the genera of the tribe Durioneae should be excluded from Bombacaceae. Most members of the erstwhile family Bombacaceae have been transferred to the subfamily Bombacoideae within the family Malvaceae [40]. This phylogenetic ordering was supported by our phylogenetic analysis of the complete chloroplast genomes of Marvel plants [41]. The estimated divergence time of *B. ceiba* and *D. zibethinus* was 29.5 million years ago, while that of *B. ceiba* and *G. arboretum* was about 20.6 million years ago (Fig. 2b).

Genes under positive selection

B. ceiba is an ecologically important plant which could survive in extreme climate conditions, such as

hot-dry valleys [7]. According to the neutral theory of molecular evolution [42], the ratio of nonsynonymous substitution rate (Ka) and synonymous substitution rate (Ks) of protein coding genes can be used to identify genes that show signatures of natural selection. We calculated average Ka/Ks values and conducted the branch-site likelihood ratio test using Codeml implemented in the PAML package [38] to identify positively selected genes in the *B. ceiba* lineage. These genes might contribute to the adaption to unfavorable environments. Thirty-six genes with signatures of positive selection were identified ($P \leq 0.05$), of which 32 genes could be annotated with potential functions in the Swissport database (Table S15). One gene is homologous to a desiccation protectant protein coding gene (*Lea14*). There is a strong association of LEA proteins with abiotic stress tolerance, particularly during dehydration and cold stress [43]. This gene could potentially contribute to the adaption of *B. ceiba* to the dry valley environment. Another gene showing signs of positive selection is homologous to the gene coding for Kelch domain-containing protein 4. The Kelch domain-containing proteins are involved in regulating major processes such as growth, development, and biotic and abiotic stress responses in plants [44, 45]. Some researchers suggested that the E3 ubiquitin-protein ligase (RFWD3) has potential roles in plant stress responses [46, 47]. Twenty-one positively selected sites were identified in the CACTIN protein coding gene. The CACTIN protein was characterized as a negative regulator of many different developmental processes, such as embryogenesis [48]. While literature reports are rare, other identified genes might also be associated with the ecological adaption of *B. ceiba*. It should be noted that this is just a preliminary analysis of the functions of these genes, further studies would be needed to clarify their roles.

Whole-genome duplication and Gene family expansion analysis

We used four-fold synonymous third-codon transversion (4DTv) estimation to detect whole-genome duplication (WGD) events in the *B. ceiba* genome. To this end, paralogous sequences of *B. ceiba*, *T. cacao*, *V. vinfira*, *S. lycopersicum* and *D. zibethinus* was identified with OrthoMCL [34]. Then, protein sequences for each of these plants were aligned against one-other with Blastp [12] (using an E-value threshold of ≤1e−5) to identify conserved paralogs in each species. Finally, potential WGD events in each genome were evaluated based on their 4DTv distribution. The WGD analysis suggested that *B. ceiba* experienced the same same WGD events as other Dicotyledons, and that *B. ceiba* and *D. zibethinus* went through their WGD events before diverging from their common ancestor (Fig. 2c).

The OrthoMCL gene family analysis results were analyzed further by using CAFE (Computational Analysis of gene Family Evolution, v3.0) [49] to detect expanded gene families. This approach revealed 5,612 expanded gene families and 1,902 contracted gene families in the *B. ceiba* lineage (Fig. S7).

Conclusion

This paper reports the sequencing, assembly, and annotation of the *B. ceiba* genome along with details of its evolutionary history. The genomic data generated in this work will be a valuable resource for further genetic improvement and effective use of the red silk cotton tree.

Availability of supporting data

The raw data from our genome project was deposited in the SRA (Sequence Read Archive) database of national center for biotechnology information with Bioproject ID PRJNA429932. The assembly and annotation of the *B. ceiba* genome and other supporting data, including BUSCO results, are available in

the GigaScience database, GigaDB [50]. Versions and main parameters of the software used in this study are provided in Table S16 in the supplementary file.

Competing interests

S. S. is an employee of Nextomics Bioscences. Other authors declare that they have no competing interests.

Authors' contributions

L. T. and B. T. designed the project; H. W., C. L. and H. C. collected samples and extracted the DNA and RNA samples; Y. G., S. S., H. W. and C. L. worked on sequencing and data analyzing; Y. G. wrote the manuscript; L. T., B. T. and D. D. revised the manuscript; All authors read and approved the final version of the manuscript.

Acknowledgements

We thank Guanglong Ou, Jianmei Wu and Renbin Zhu for offering photos of *B. ceiba*. This study was financially supported by the National Science Foundation of China (grant 31460561, 31760103, 31460179 and 31660680), the Key Laboratory of Forest Resources Conservation and Utilization in the Southwest Mountains of China (Southwest Forestry University), Ministry of Education, and the Yunnan Applied Basic Research Project (grant 2017FD145).

References

1. Barwick M. Tropical and Subtropical Trees. Portland, OR: Timber Press; 2004.
2. Jain V , Verma SK. Pharmacology of *Bombax Ceiba* Linn. Berlin Heidelberg: Springer; 2012.
3. Chand S , Singh AK. In Vitro Propagation of *Bombax Ceiba* L. (Silkcotton). Silvae Genetica. 1999;48 (6):313-7.
4. Nair GS , Bai Y. Ethnobotanical Value of Dry, Fallen Ovaries of *Bombax Ceiba* L. (Bombacaceae: Malvales). Journal of Threatened Taxa. 2012;4 (15):3443-6.
5. Ngwuluka NC. Are *Bombax Buonopozense* and *Bombax Malabaricum* Possible Nutraceuticals for Age Management? Preventive Medicine. 2012;54 (S3):64-70.
6. Pankaj HC , Somshekhar SK. *Bombax Ceiba* Linn.: Pharmacognosy, Ethnobotany and Phyto-Pharmacology. Pharmacognosy Communications. 2012;2 (3):2-9.

7. Zhou Z, Ma H, Lin K, et al. RNA-Seq Reveals Complicated Transcriptomic Responses to Drought Stress in a Nonmodel Tropic Plant, *Bombax Ceiba* L. Evolutionary Bioinformatics. 2015;11 (S1):27-37.
8. Peng C, Wen D, Sun Z, et al. Response of Some Plants for Municipal Greening to Air Pollutants. Journal of Tropical and Subtropical Botany. 2002;10 (4):321-7.
9. Elhagrassi AM, Ali MM, Osman AF, et al. Phytochemical Investigation and Biological Studies of *Bombax Malabaricum* Flowers. Natural Product Research. 2011;25 (2):141-51.
10. Marçais G , Kingsford C. A Fast, Lock-Free Approach for Efficient Parallel Counting of Occurrences of K-Mers. Bioinformatics. 2011;27 (6):764-70.
11. Chaisson MJ , Tesler G. Mapping Single Molecule Sequencing Reads Using Basic Local Alignment with Successive Refinement (Blasr): Application and Theory. BMC Bioinformatics. 2012;13 (1):238.
12. Camacho C, Coulouris G, Avagyan V, et al. Blast+: Architecture and Applications. BMC Bioinformatics. 2009;10 (1):421.
13. Worley KC, English AC, Richards S, et al. Improving Genomes Using Long Reads and Pbjelly 2. In: *International Plant and Animal Genome Conference Xxii* 2014.
14. Li H , Durbin R. Fast and Accurate Short Read Alignment with Burrows–Wheeler Transform. Oxford University Press; 2009.
15. Walker BJ, Abeel T, Shea T, et al. Pilon: An Integrated Tool for Comprehensive Microbial Variant Detection and Genome Assembly Improvement. Plos One. 2014;9 (11):e112963.
16. Kim D, Langmead B , Salzberg SL. Hisat: A Fast Spliced Aligner with Low Memory Requirements. Nature Methods. 2015;12 (4):357-60.
17. Simão FA, Waterhouse RM, Ioannidis P, et al. Busco: Assessing Genome Assembly and Annotation Completeness with Single-Copy Orthologs. Bioinformatics. 2015;31 (19):3210-2.
18. Thiel T, Michalek W, Varshney RK, et al. Exploiting Est Databases for the Development and Characterization of Gene-Derived Ssr-Markers in Barley (*Hordeum Vulgare* L.). Theoretical and Applied Genetics. 2003;106 (3):411-22.
19. Tarailograovac M , Chen N. Using Repeatmasker to Identify Repetitive Elements in Genomic Sequences. 2009;3:4-14.
20. Bao W, Kojima KK , Kohany O. Repbase Update, a Database of Repetitive Elements in Eukaryotic Genomes. Mobile DNA. 2015;6 (1):11.
21. Thomas N, Martis MM, Roessner SK, et al. Mips Plantsdb: A Database Framework for Comparative Plant Genome Research. Nucleic Acids Research. 2013;41:1144-51.
22. Kalvari I, Argasinska J, Quinones-Olvera N, et al. Rfam 13.0: Shifting to a Genome-Centric Resource for Non-Coding RNA Families. Nucleic Acids Research. 2017; doi:https://doi.org/10.1093/nar/gkx1038.
23. Lowe TM , Eddy SR. Trnascan-Se: A Program for Improved Detection of Transfer RNA Genes in Genomic Sequence. Nucleic Acids Research. 1997;25 (5):955-64.
24. Lagesen K, Hallin P, Rødland EA, et al. RNAmmer: Consistent and Rapid Annotation of Ribosomal Rna Genes. Nucleic Acids Research. 2007;35 (9):3100-8.
25. Birney E , Durbin R. Using Genewise in the Drosophila Annotation Experiment. Genome Research. 2000;10 (4):547-8.
26. Stanke M, Steinkamp R, Waack S, et al. Augustus: A Web Server for Gene Finding in Eukaryotes. Nucleic Acids Research. 2004;32:309-12.

27. Blanco E, Parra G , Guigó R. Using Geneid to Identify Genes. Current protocols in bioinformatics. 2007; 4 (3):1-28.
28. Majoros WH, Pertea M , Salzberg SL. Tigrscan and Glimmerhmm: Two Open Source Ab Initio Eukaryotic Gene-Finders. Bioinformatics. 2004;20 (16):2878-9.
29. Bromberg Y , Rost B. Snap: Predict Effect of Non-Synonymous Polymorphisms on Function. Nucleic Acids Research. 2007;35 (11):3823-35.
30. Haas BJ, Salzberg SL, Wei Z, et al. Automated Eukaryotic Gene Structure Annotation Using Evidencemodeler and the Program to Assemble Spliced Alignments. Genome Biology. 2008;9 (1):R7.
31. Moriya Y, Itoh M, Okuda S, et al. Kaas: An Automatic Genome Annotation and Pathway Reconstruction Server. Nucleic Acids Research. 2007;35:W182-W5.
32. Quevillon E, Silventoinen V, Pillai S, et al. Interproscan: Protein Domains Identifier. Nucleic Acids Research. 2005;33:116-20.
33. M GD, Shengqiang S, Russell H, et al. Phytozome: A Comparative Platform for Green Plant Genomics. Nucleic acids research. 2012;40 (Database issue):D1178-D86.
34. Li L, Stoeckert CJ , Roos DS. Orthomcl: Identification of Ortholog Groups for Eukaryotic Genomes. Genome Research. 2003;13 (9):2178-89.
35. Edgar RC. Muscle: Multiple Sequence Alignment with High Accuracy and High Throughput. Nucleic Acids Research. 2004;32 (5):1792-7.
36. Talavera G , Castresana J. Improvement of Phylogenies after Removing Divergent and Ambiguously Aligned Blocks from Protein Sequence Alignments. Systematic Biology. 2007;56 (4):564-77.
37. Stamatakis A. Raxml Version 8: A Tool for Phylogenetic Analysis and Post-Analysis of Large Phylogenies. Bioinformatics. 2014;30 (9):1312-3.
38. Yang Z. Paml 4: Phylogenetic Analysis by Maximum Likelihood. Molecular Biology and Evolution. 2007;24 (8):1586-91.
39. Baum DA, Smith DW, Yen A, et al. Phylogenetic Relationships of Malvatheca (Bombacoideae and Malvoideae; Malvaceae Sensu Lato) as Inferred from Plastid DNA Sequences. American Journal of Botany. 2004;91 (11):1863-71.
40. Heywood, V.H, Brummitt, et al. Flowering Plant Families of the World. Richmond, Surrey: Royal Botanic Gardens; 2007.
41. Gao Y, Wang H, Liu C, et al. Complete Chloroplast Genome Sequence of the Red Silk Cotton Tree (*Bombax Ceiba*). Mitochondrial DNA Part B. 2018;3 (1):315-6. doi:10.1080/23802359.2017.1422399.
42. Kimura M: The neutral theory of molecular evolution. Cambridge, England: Cambridge University Press 1983
43. Shinde S, Nurul IM , Ng CK. Dehydration Stress-Induced Oscillations in LEA Protein Transcripts Involves Abscisic Acid in the Moss, *Physcomitrella Patens*. New Phytologist. 2012;195 (2):321-8.
44. Feder A, Burger J, Gao S, et al. Focus on Metabolism: A Kelch Domain-Containing F-Box Coding Gene Negatively Regulates Flavonoid Accumulation in Muskmelon. Plant Physiology. 2015;169 (3):1714-26.
45. Zhang X, Gou M, Guo C, et al. Down-Regulation of Kelch Domain-Containing F-Box Protein in Arabidopsis Enhances the Production of (Poly)Phenols and Tolerance to Ultraviolet

Radiation. Plant Physiology. 2015;167 (2):337-50.

46. Serrano I, Campos L , Rivas S. Roles of E3 Ubiquitin-Ligases in Nuclear Protein Homeostasis During Plant Stress Responses. Frontiers in Plant Science. 2018;9 (139) doi:10.3389/fpls.2018.00139.

47. Duplan V , Rivas S. E3 Ubiquitin-Ligases and Their Target Proteins During the Regulation of Plant Innate Immunity. Frontiers in Plant Science. 2014;5 (42) doi:10.3389/fpls.2014.00042.

48. Baldwin KL, Dinh EM, Hart BM, et al. CACTIN is an Essential Nuclear Protein in *Arabidopsis* and may be Associated with the Eukaryotic Spliceosome. Febs Letters. 2013;587 (7):873-9.

49. De Bie T, Cristianini N, Demuth JP, et al. Cafe: A Computational Tool for the Study of Gene Family Evolution. Bioinformatics. 2006;22 (10):1269-71.

50. Gao Y, Wang H, Liu C, Chu H, Dai D, Song S, et al. Supporting data for "De novo genome assembly of the red silk cotton tree (Bombax ceiba)". GigaScience Database 2018. http://dx.doi.org/10.5524/100445

Figure 1. Example of the red silk cotton tree (*B. ceiba*). (a) Natural habitat of *B. ceiba* (image from Guanglong Ou). (b) *B. ceiba* used as municipal greening trees (image from Jianmei Wu). (c) The flower of *B. ceiba* (image from Renbin Zhu).

Figure 2. Phylogenetic relationships and genomic comparisons between *B. ceiba* and other plants. (a) A Venn diagram of shared gene families between *B. ceiba* and three other Malvales plants, with *A. thaliana* as an outgroup. Each number represents a gene family number. (b) Inferred phylogenetic tree across 13 plant species. The estimated divergence time (Mya) is shown at each node. (c) WGD events of four plants (*B. ceiba*, *D. zibethinus*, *S. lycopersicum* and *V. vinifera*) inferred by 4DTv estimations. Peaks corresponding to speciation, recent and ancient WGDs are indicated by arrows.

Additional files

Figure S1. Frequency distribution of the 17-mer graph analysis used to estimate the size of the *B. ceiba* genome.

Figure S2. GC content distribution of the *B. ceiba* genome. The GC content was established using 500 bp sliding windows.

Figure S3. The GC depth distribution of the *B. ceiba* genome.

Figure S4. Comparison of gene structure characteristics in *B. ceiba* to that in other plants. a, CDS length; b, Exon length; c, Exon number; d, Gene length; e, Intron length.

Figure S5. Gene orthology determined by comparing genomes using the OrthoMCL software.

Figure S6. The maximum-likelihood phylogeny of *B. ceiba* and 13 other plants.

Figure S7. Gene family expansions and contractions in *B. ceiba* and 13 other plants.

Table S1. Sequencing statistics from the PacBio platform

Table S2. Summary of the transcriptomes and their mapping rates on the genome assembly

Table S3. Estimation of genome size based on 17-mer statistics

Table S4. Blast results of *Bombax ceiba* genome against the NCBI Nt database

Table S5. Summary of the BioNano optical mapping data

Table S6. Summary of the final genome assembly

Table S7. Summary of BUSCO analysis results

Table S8. Summary of the SSR search results

Table S9. Repeat annotation of the *Bombax ceiba* genome assembly

Table S10. Summary of non-protein-coding gene annotations in the *Bombax ceiba* genome assembly

Table S11. Gene annotation statistics of the *Bombax ceiba* genome assembly

Table S12. Comparative gene statistics

Table S13. Functional annotation of predicted genes of *Bombax ceiba*

Table S14. Summary statistics of gene families in 13 plant species

Table S15. Candidate positively selected genes in the *Bombax ceiba* lineage

Table S16. Versions and main parameters of the software used in this study

Figure 1

Click here to download Figure renamed_51f32.tif

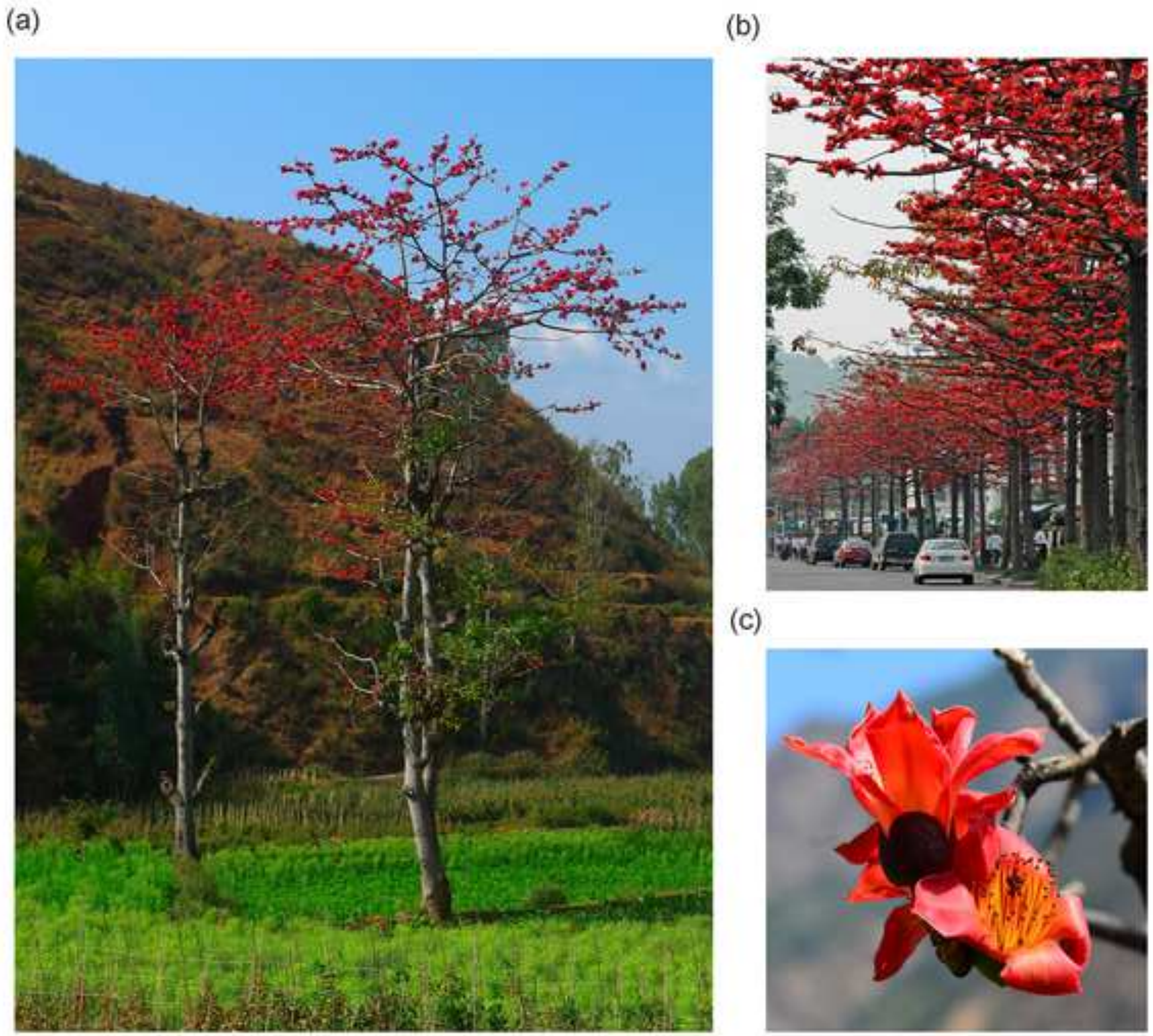

Figure 2

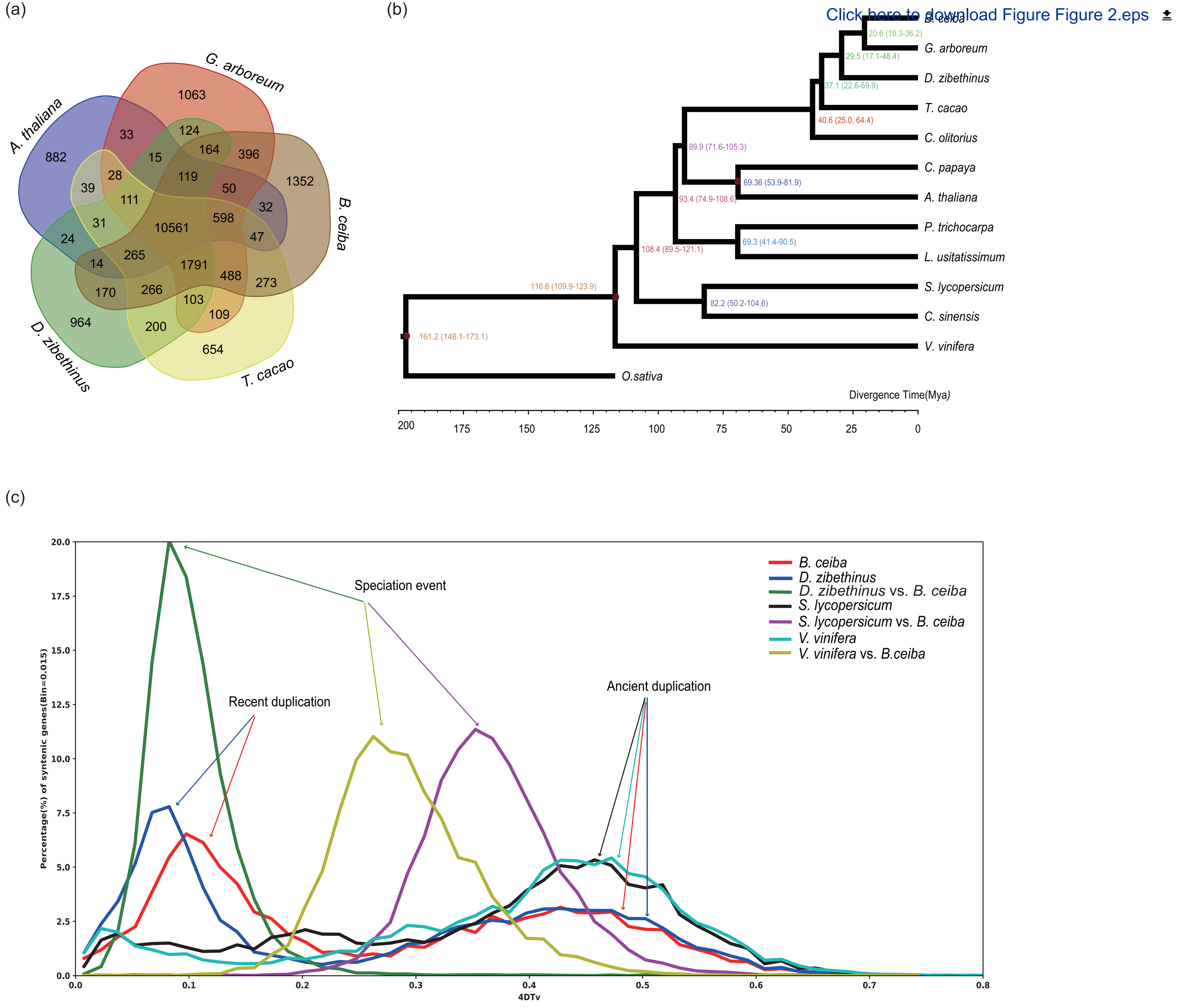

Click here to download Figure Figure 2.eps

Supplementary Material

Click here to access/download
Supplementary Material
Supplementary file20180403.docx
