# Supplementary material for: De novo genome assembly of the red silk cotton tree (Bombax ceiba)
Source: Gigascience. 2018 May 10;7(5):giy051. doi: 10.1093/gigascience/giy051 (PMC5967522; doi:10.1093/gigascience/giy051)
Supplement: Supplemental material [file giy051_supp.docx]

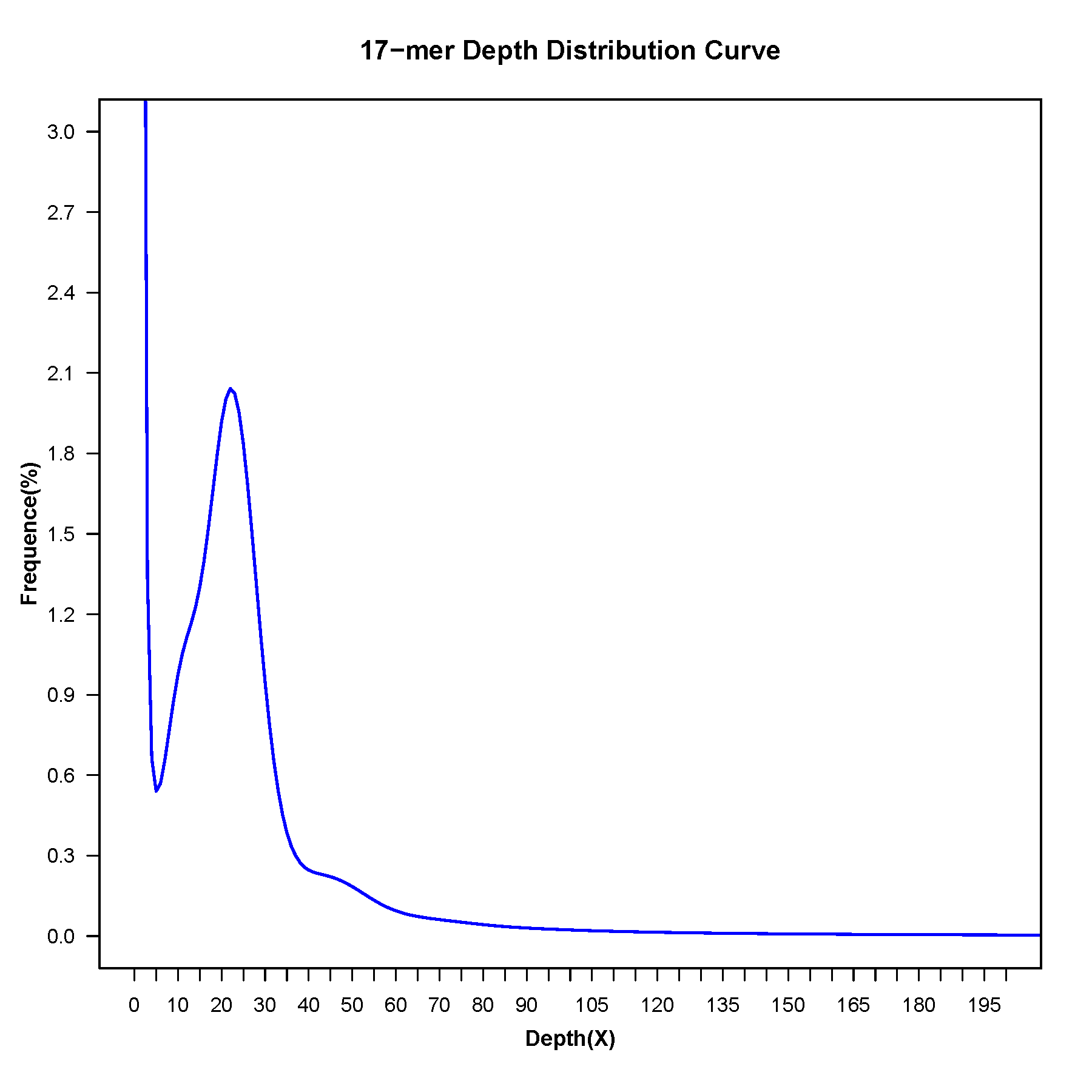


**Figure S1. Frequency distribution of the 17-mer graph analysis uesd to estimate the size of the *B. ceiba* genome.**


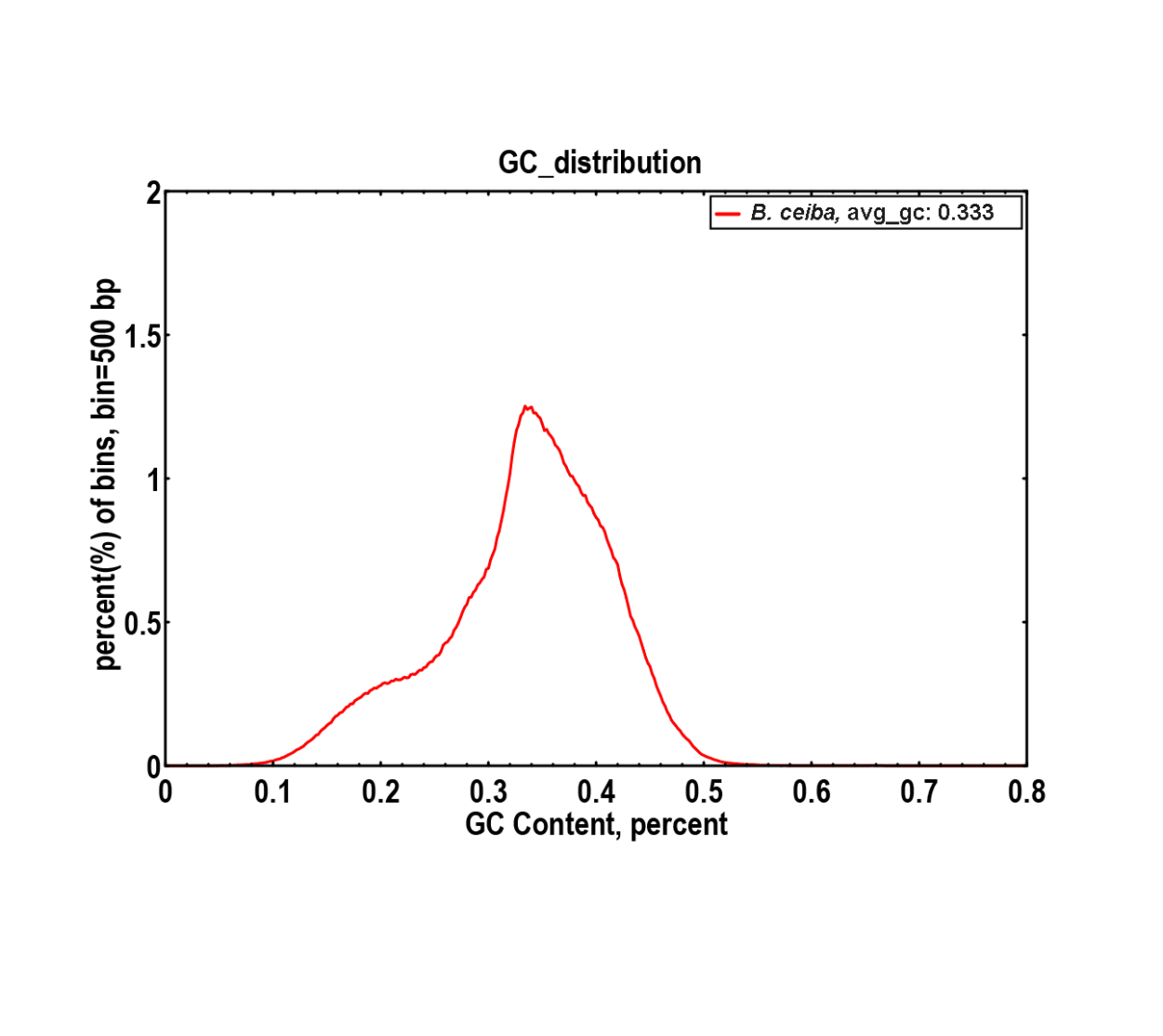


**Figure S2. GC content distribution of the *B. ceiba* genome. The GC content was established using 500 bp sliding windows.**


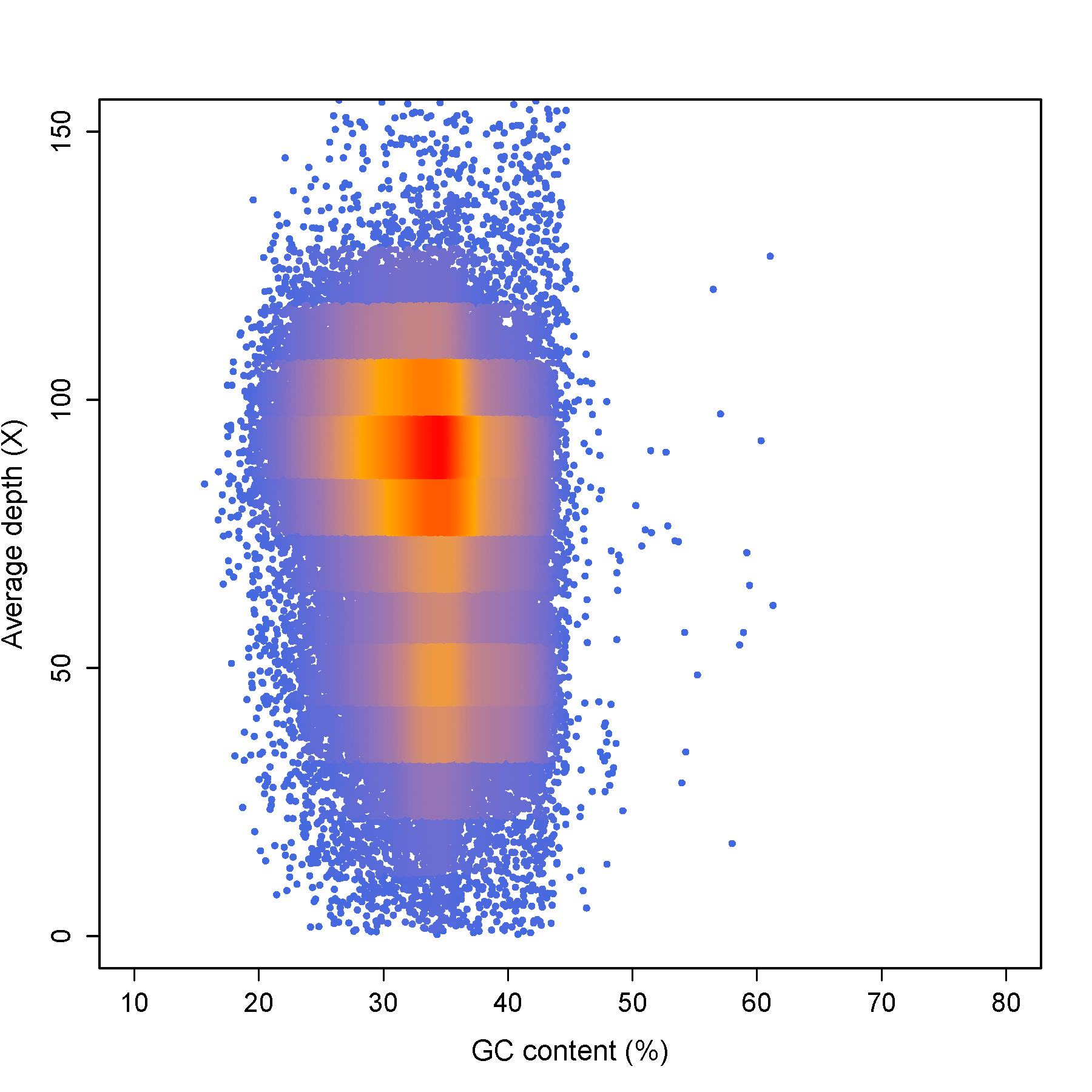


**Figure S3. The GC depth distribution of the *B. ceiba* genome.**


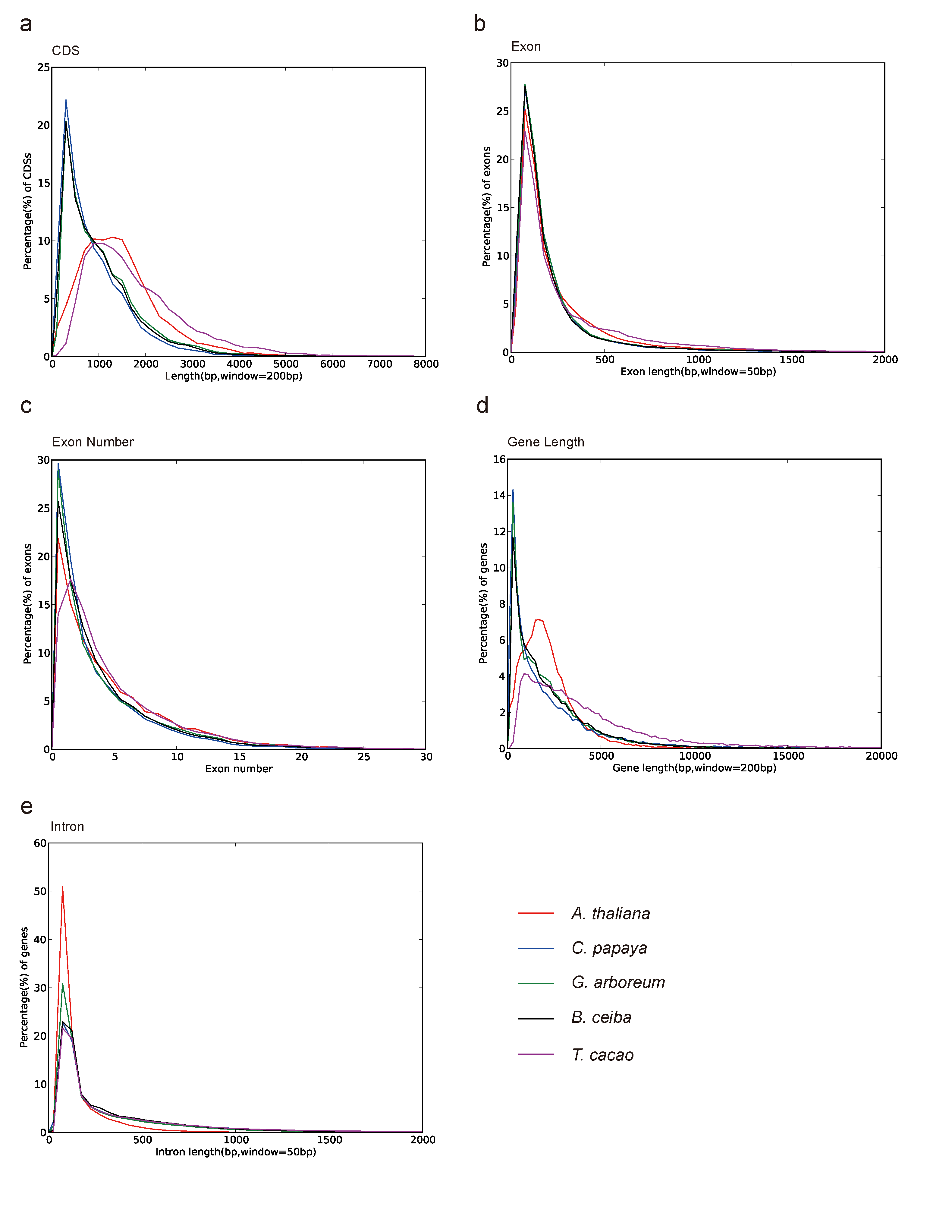


**Figure S4. Comparison of gene structure characteristics in *B. ceiba* to that in other plants. a, CDS length; b, Exon length; c, Exon number; d, Gene length; e, Intron length.**


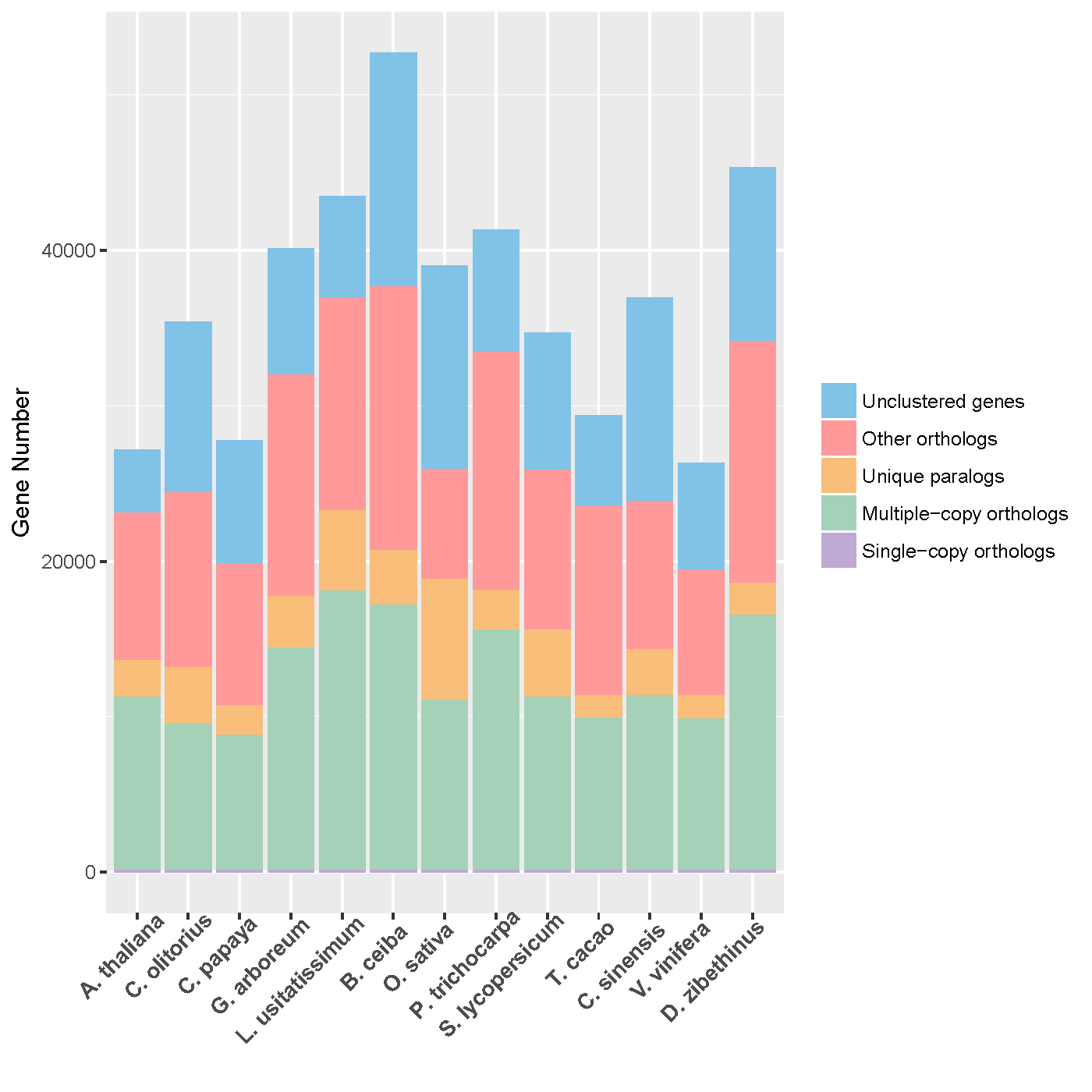


**Figure S5. Gene orthology determined by comparing genomes using the OrthoMCL software.**


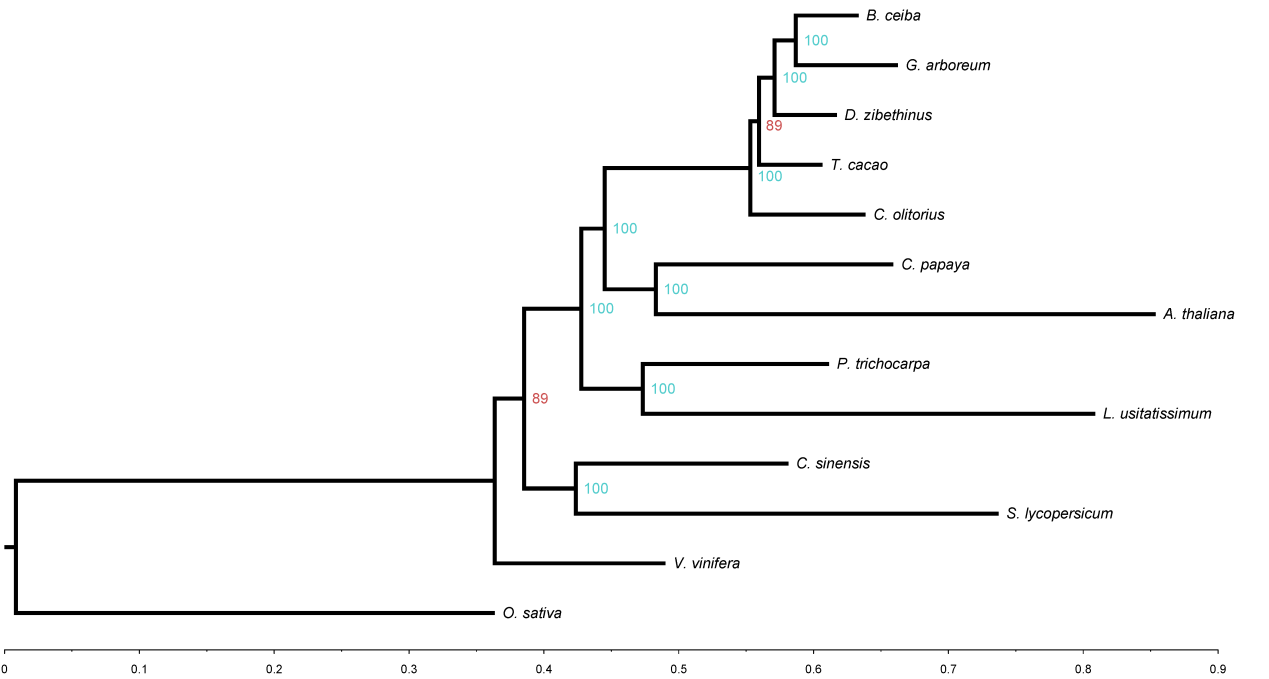


**Figure S6. The maximum-likelihood phylogeny of *B. ceiba* and 13 other plants. The bootstrap value is shown at each node.**

**
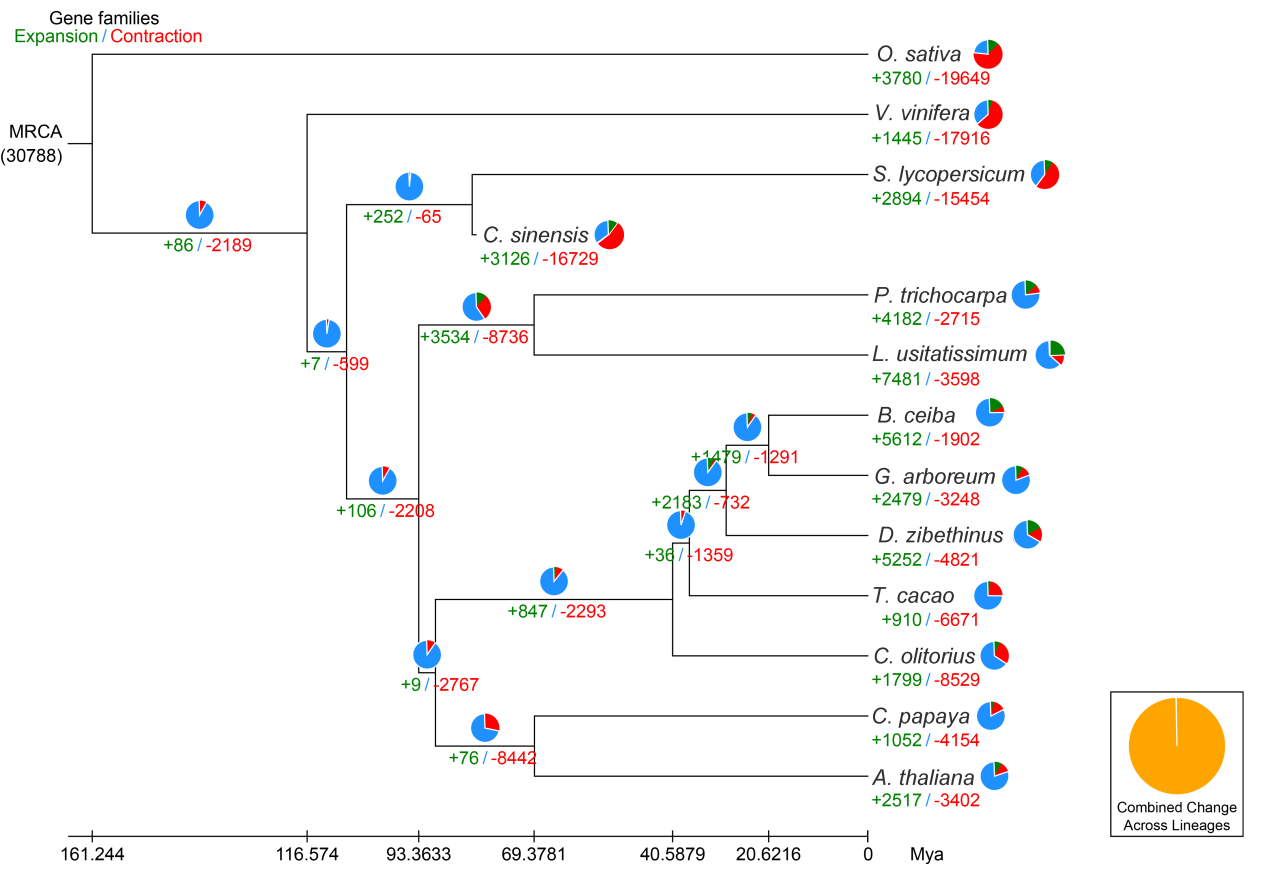
**

**Figure S7. Gene family expansions and contractions in *B. ceiba* and 13 other plants.**

| **Table S1. Sequencing statistics from the PacBio platform** | | | |
| --- | --- | --- | --- |
| Cells | Subreads Mean Length (Kb) | Subreads N50 (Kb) | Subreads Read Base (Gb) |
| 19 | 8.4 | 11.9 | 86.0 |

| **Table S2. Summary of the transcriptomes and their mapping rate on the genome assembly** | | | | |
| --- | --- | --- | --- | --- |
| Sample | Tissue | Total Reads Number | Mapped Reads Number | Mapping Rate (%) |
| BC4_1 | calyx | 40,915,018 | 36276595 | 88.66 |
| BC4_2 | petal | 33,985,798 | 30125571 | 88.64 |
| BC4_3 | stamen | 38,296,878 | 34880799 | 91.08 |
| BC4_4 | pistil | 40,497,408 | 36240029 | 89.49 |
| BC4_5 | bark | 30,946,774 | 26235919 | 84.78 |
| BC5_1 | calyx | 51,191,192 | 47205453 | 92.21 |
| BC6_1 | bud | 29,441,936 | 25167463 | 85.48 |
| BC6_3 | root | 30,816,034 | 26132972 | 84.8 |

| **Table S3. Estimation of genome size based on 17-mer statistics** | | | | | | |
| --- | --- | --- | --- | --- | --- | --- |
| K-mer Value | K-mer Number | K-mer Depth | Genome Size (Gb) | Used Bases (Gb) | Used Reads | Heterozygosity Rate (%) |
| 17 | 17,801,654,802 | 22 | 0.809 | 20 | 138,025,302 | 0.88 |

| **Table S4. Blast results of *Bombax ceiba* genome against the NCBI Nt database** | | |
| --- | --- | --- |
| Species | Blast_hits | Plant |
| *Theobroma cacao* | 1181 | Yes |
| *Gossypium hirsutum* | 223 | Yes |
| *Gossypium arboreum* | 200 | Yes |
| *Gossypium raimondii* | 97 | Yes |
| *Gossypium trilobum* | 12 | Yes |
| *Gossypium nelsonii* | 5 | Yes |
| *Gossypium davidsonii* | 5 | Yes |
| *Gossypium barbadense* | 3 | Yes |
| *Gossypium anomalum* | 2 | Yes |
| *Gossypium populifolium* | 1 | Yes |
| *Gossypium longicalyx* | 1 | Yes |
| *Gossypium herbaceum* | 1 | Yes |
| *Gossypium aridum* | 1 | Yes |
| *Gossypium areysianum* | 1 | Yes |
| *Vitis vinifera* | 242 | Yes |
| *Citrus sinensis* | 180 | Yes |
| *Citrus medica* | 2 | Yes |
| *Tilia amurensis* | 33 | Yes |
| *Glycine max* | 28 | Yes |
| *Glycine tomentella* | 1 | Yes |
| *Tilia paucicostata* | 27 | Yes |
| *Bombax ceiba* | 26 | Yes |
| *Populus trichocarpa* | 25 | Yes |
| *Populus euphratica* | 2 | Yes |
| *Vigna angularis* | 24 | Yes |
| *Lotus japonicus* | 22 | Yes |
| *Ipomoea nil* | 19 | Yes |
| *Corchorus capsularis* | 19 | Yes |
| *Hibiscus syriacus* | 12 | Yes |
| *Medicago truncatula* | 11 | Yes |
| *Gossypioides kirkii* | 9 | Yes |
| *Ricinus communis* | 5 | Yes |
| *Cucumis melo* | 5 | Yes |
| *Cicer arietinum* | 5 | Yes |
| *Vitis hybrid cultivar* | 3 | Yes |
| *Tilia oliveri* | 3 | Yes |
| *Solanum pennellii* | 3 | Yes |
| *Arabis alpina* | 8 | Yes |
| *Arachis ipaensis* | 3 | Yes |
| *Arachis duranensis* | 3 | Yes |
| *Arabidopsis thaliana* | 3 | Yes |
| *Theobroma grandiflorum* | 2 | Yes |
| *Talipariti hamabo* | 2 | Yes |
| ***Psyllidae* sp.** | **2** | **No** |
| *Lilium tsingtauense* | 2 | Yes |
| *Carica papaya* | 2 | Yes |
| *Althaea officinalis* | 2 | Yes |
| *Adansonia digitata* | 2 | Yes |
| *Vigna riukiuensis* | 1 | Yes |
| *Vigna minima* | 1 | Yes |
| *Triticum dicoccoides* | 1 | Yes |
| ***Trioza eugeniae*** | **1** | **No** |
| *Spondias tuberosa* | 1 | Yes |
| *Salix alba* | 1 | Yes |
| *Pterospora andromedea* | 1 | Yes |
| *Phoenix dactylifera* | 1 | Yes |
| *Oryza officinalis* | 1 | Yes |
| *Olea europaea* | 1 | Yes |
| *Musa acuminata* | 1 | Yes |
| *Metzgeria pubescens* | 1 | Yes |
| *Matthiola incana* | 1 | Yes |
| *Lupinus angustifolius* | 1 | Yes |
| *Lens culinaris* | 1 | Yes |
| *Juglans regia* | 1 | Yes |
| *Gonystylus bancanus* | 1 | Yes |
| *Fragaria vesca* subsp.vesca | 1 | Yes |
| *Elaeis guineensis* | 1 | Yes |
| ***Diptacus* sp.** | **1** | **No** |
| ***Dichorragia nesimachus*** | **1** | **No** |
| *Capsicum annuum* | 1 | Yes |
| *Brassica napus* | 1 | Yes |
| *Adansonia madagascariensis* | 1 | Yes |
|  |  |  |
| Total | 2494 | - |

| **Table S5. Data summary of Bionano optical mapping** | | | |
| --- | --- | --- | --- |
| Data type | Quantity (Gb) | Average Label Density (per 100Kb) | N50 (Kb) |
| Raw data | 206.1 | 6.2 | 227.8 |
| Clean data | 160.0 | 6.2 | 269.9 |

| **Table S6. Summary of the genome assembly** | | | | | |
| --- | --- | --- | --- | --- | --- |
| Strategy | StatType | Scaffold Length（bp） | Scaffold Number | Contig Length（bp） | Contig Number |
| PacBio assembly |  |  |  |  |  |
|  | N50 | - | - | 726,751 | 325 |
|  | N60 | - | - | 555,672 | 459 |
|  | N70 | - | - | 402,875 | 640 |
|  | N80 | - | - | 259,717 | 899 |
|  | N90 | - | - | 125,909 | 1,357 |
|  | Longest | - | - | 3,986,692 | 1 |
|  | Total | - | - | 851,604,865 | 3,629 |
|  | Length≥1kb | - | - | 851,604,865 | 3,629 |
|  | Length≥2kb | - | - | 851,558,783 | 3,597 |
|  | Length≥5kb | - | - | 851,073,390 | 3,463 |
| BioNano optical scaffolding |  |  |  |  |  |
|  | N50 | 2,064,896 | 125 | 1,023,644 | 229 |
|  | N60 | 1,499,392 | 175 | 761,718 | 328 |
|  | N70 | 1,036,866 | 246 | 546,659 | 464 |
|  | N80 | 555,187 | 364 | 349,177 | 663 |
|  | N90 | 163,167 | 658 | 143,718 | 1,052 |
|  | Longest | 9,553,305 | 1 | 5,730,937 | 1 |
|  | Total | 894,504,361 | 2,759 | 869,109,142 | 3,145 |
|  | Length≥1kb | 894,504,361 | 2,759 | 869,109,142 | 3,145 |
|  | Length≥2kb | 894,462,192 | 2,730 | 869,066,973 | 3,116 |
|  | Length≥5kb | 894,031,831 | 2,610 | 868,636,612 | 2,996 |

| **Table S7. Summary of BUSCO analysis results** | | |
| --- | --- | --- |
| Type | Number | Percent (%) |
| Complete BUSCOs (C) | 1,358 | 94.4 |
| Complete and single-copy BUSCOs (S) | 1,111 | 77.2 |
| Complete and duplicated BUSCOs (D) | 247 | 17.2 |
| Fragmented BUSCOs (F) | 19 | 1.3 |
| Missing BUSCOs (M) | 63 | 4.3 |
| Total BUSCO groups searched | 1,440 | - |

| **Table S8. Summary of the SSR search results** | | |
| --- | --- | --- |
| Repeat type | Unit size (repeat number) | Number |
| mono-nucleotide | 1 (≥10) | 310,369 |
| Di-nucleotide | 2 (≥6) | 105,004 |
| Tri-nucleotide | 3 (≥5) | 30,925 |
| Tetra-nucleotide | 4 (≥5) | 6,448 |
| Penta-nucleotide | 5 (≥5) | 1,165 |
| Hexa-nucleotide | 6 (≥5) | 524 |

| **Table S9. Repeat annotation of the *Bombax ceiba* genome assembly** | | | | | | | | |
| --- | --- | --- | --- | --- | --- | --- | --- | --- |
| Type | Repbase TEs | | TE proteins | | RepeatModeler | | Combined TEs | |
|  | Length (bp) | % in genome | Length (bp) | % in genome | Length (bp) | % in genome | Length (bp) | % in genome |
| DNA | 6,289,990 | 0.72 | 3,774,898 | 0.43 | 6820135 | 0.78 | 11746145 | 1.35 |
| LINE | 1,421,160 | 0.16 | 2,466,943 | 0.28 | 1323663 | 0.15 | 3429439 | 0.39 |
| LTR | 185,631,223 | 21.36 | 152,784,879 | 17.58 | 353960994 | 40.73 | 415969672 | 47.86 |
| SINE | 12,345 | ~0 | 0 | 0 | 10240 | ~0 | 21116 | ~0 |
| Other | 1,635,560 | 0.19 | 7,646 | ~0 | 0 | 0 | 57727611 | 6.65 |
| Unknown | 58,066 | 0.01 | 0 | 0 | 59264082 | 6.82 | 35172901 | 4.05 |
| Total | 195,048,344 | 22.44 | 159,034,366 | 18.3 | 420329511 | 48.36 | 524066884 | 60.30 |
| DNA, DNA transposon; LINE, long interspersed nuclear element; TEs, transposable elements; | | | | | | | | |
| SINE, short interspersed nuclear element; LTR, long terminal repeat. | | | | | | | | |

| **Table S10. Summary of non-protein-coding gene annotations in the *Bombax ceiba* genome assembly** | | | | |
| --- | --- | --- | --- | --- |
| Type | Copy Number | Average Length (bp) | Total Length (bp) | Percentage (%) of Genome |
| **rRNA** | **6,772** | **193.85** | **1,312,724** | **0.146754** |
| 18S | 72 | 1,865.78 | 134,336 | 0.015018 |
| 28S | 72 | 5,805.82 | 418,019 | 0.046732 |
| 5.8S | 69 | 155.51 | 10,730 | 0.0012 |
| 5S | 6,628 | 114.72 | 760,369 | 0.085005 |
| **snRNA** | **727** | **114.86** | **83,501** | **0.009335** |
| CD-box | 532 | 107.03 | 56,939 | 0.006365 |
| HACA-box | 65 | 121.12 | 7,873 | 0.00088 |
| splicing | 130 | 143.76 | 18,689 | 0.002089 |
| **miRNA** | **496** | **126.56** | **62,773** | **0.007018** |
| **tRNA** | **894** | **74** | **66,449** | **0.007429** |

| **Table S11. Gene annotation statistics of the *Bombax ceiba* genome assembly** | | | | | | | |
| --- | --- | --- | --- | --- | --- | --- | --- |
| Methods | | Total Number | Average Gene Length (bp) | Average CDS Length (bp) | Average Exons Number per Gene | Average Exon Length (bp) | Average Intron Length (bp) |
| Homolog | *A. thaliana* | 51,180 | 2,214.92 | 986.58 | 3.71 | 266.16 | 453.81 |
|  | *C. papaya* | 76,572 | 1,427.15 | 699.41 | 2.79 | 250.7 | 406.6 |
|  | *G. um arboreum* | 99,385 | 2,026.02 | 806.82 | 3.09 | 260.84 | 582.47 |
|  | *T. cacao* | 59,779 | 3,054.79 | 1,246.19 | 4.27 | 292 | 553.47 |
| *De novo* | Augustus | 61,025 | 2,660.02 | 1,025.63 | 5.17 | 198.24 | 391.59 |
|  | GeneID | 45,787 | 5,022.61 | 1,041.88 | 5.13 | 203.29 | 964.98 |
|  | GlimmerHMM | 44,854 | 2,308.06 | 988.07 | 4.17 | 237.16 | 416.89 |
|  | SNAP | 64,334 | 1,404.82 | 732.72 | 3.42 | 214.33 | 277.88 |
| EVidenceModeler | | 52,705 | 2,418.37 | 1,019.38 | 4.57 | 222.9 | 391.52 |

| **Table S12. Comparative gene statistics** | | | | | | |
| --- | --- | --- | --- | --- | --- | --- |
| Gene set | Number | Average Gene Length (bp) | Average CDS Length (bp) | Average Exons Number per Gene | Average Exon Length (bp) | Average Intron Length (bp) |
| *T. cacao* | 29,390 | 5,746 | 1,833 | 5.5 | 333.59 | 870.19 |
| *G. arboreum* | 40,134 | 2,414 | 1,089 | 4.61 | 236.4 | 367.41 |
| *A. thaliana* | 27,173 | 2,197 | 1,487 | 5.34 | 278.73 | 163.58 |
| *C. papaya* | 27,725 | 2,360 | 894 | 4.06 | 220.24 | 479.44 |
| *B. ceiba* | 52,705 | 2,418 | 1,019 | 4.57 | 222.9 | 391.52 |

| **Table S13. Functional annotation of predicted genes of *Bombax ceiba*** | | | |
| --- | --- | --- | --- |
|  | Database | Number | Percent (%) |
| **Annotated** |  | **47,105** | **89.37** |
|  | KEGG | 14,379 | 27.28 |
|  | InterProScan | 36,135 | 68.56 |
|  | GO | 26,100 | 49.52 |
|  | Swissprot | 36,667 | 69.57 |
|  | TrEMBL | 46,842 | 88.88 |
| **Unannotated** |  | **5600** | **10.63** |
| **Total** |  | **52,705** | **100%** |

| **Table S14. Summary statistics of gene families in 13 plant species** | | | | | | |
| --- | --- | --- | --- | --- | --- | --- |
| Species | Gene Number | Gene Number in Families | Unclustered Gene Number | Family Number | Unique Family Number | Average Gene Number per Family |
| *B. ceiba* | 52,705 | 37,736 | 14,969 | 16,586 | 906 | 2.28 |
| *D. zibethinus* | 45,324 | 34,202 | 11,122 | 14,922 | 627 | 2.29 |
| *G. arboreum* | 40,134 | 32,030 | 8,104 | 15,753 | 703 | 2.03 |
| *T. cacao* | 29,391 | 23,597 | 5,794 | 15,564 | 322 | 1.52 |
| *C. olitorius* | 35,404 | 24,456 | 10,948 | 15,205 | 980 | 1.61 |
| *C. papaya* | 27,775 | 19,898 | 7,877 | 13,510 | 479 | 1.47 |
| *C. sinensis* | 36,951 | 23,915 | 13,036 | 13,206 | 859 | 1.81 |
| *L. usitatissimum* | 43,484 | 36,966 | 6,518 | 14,021 | 1,545 | 2.64 |
| *A. thaliana* | 27,173 | 23,171 | 4,002 | 12,849 | 636 | 1.8 |
| *P. trichocarpa* | 41,335 | 33,484 | 7,851 | 14,991 | 827 | 2.23 |
| *S. lycopersicum* | 34,726 | 25,902 | 8,824 | 14,065 | 961 | 1.84 |
| *V. vinifera* | 26,346 | 19,520 | 6,826 | 13,019 | 564 | 1.5 |
| *O. sativa* | 39,044 | 25,967 | 13,077 | 12,728 | 1,987 | 2.04 |

**Table S15. Candidate positively selected genes in the *Bombax ceiba* lineage**

| *Bombax ceiba* Gene ID | P-value | Postive site number | Gene name | Swissprot function |
| --- | --- | --- | --- | --- |
| evm.model.Scaffold223.387 | 9.97E-04 | 5 | NS | NS |
| evm.model.Scaffold49.32 | 1.70E-04 | 2 | *At1g10310* | NADPH-dependent pterin aldehyde reductase |
| evm.model.Scaffold48.540 | 1.55E-04 | 1 | *APC2* | Anaphase-promoting complex subunit 2 |
| evm.model.Scaffold2236.4 | 1.28E-02 | 1 | *RAP* | RAP domain-containing protein |
| evm.model.Scaffold66.146 | 6.36E-03 | 1 | *RP1* | Pyruvate, phosphate dikinase regulatory protein 1, chloroplastic |
| evm.model.Scaffold132.264 | 8.10E-03 | 1 | *GGB* | Geranylgeranyl transferase type-1 subunit beta |
| evm.model.Scaffold140.244 | 1.59E-02 | 1 | *ABCB29* | ABC transporter B family member 29, chloroplastic |
| evm.model.Scaffold287.14 | 1.13E-03 | 1 | *At1g72550* | Phenylalanine--tRNA ligase beta subunit, cytoplasmic |
| evm.model.Scaffold60.205 | 8.16E-14 | 6 | *HMA1* | Probable cadmium/zinc-transporting ATPase HMA1 |
| evm.model.Scaffold500.47 | 7.57E-03 | 2 | *SF3A2* | Splicing factor 3A subunit 2 |
| evm.model.Scaffold1.499 | 2.49E-04 | 1 | *CRSH* | Probable GTP diphosphokinase CRSH, chloroplastic |
| evm.model.Scaffold71.292 | 2.31E-09 | 4 | *VPS9A* | Vacuolar protein sorting-associated protein 9A |
| evm.model.Scaffold426.21 | 1.56E-02 | 1 | *RPS1* | 30S ribosomal protein S1, chloroplastic |
| evm.model.Scaffold36.560 | 1.68E-02 | 1 | *Lea14* | Desiccation protectant protein Lea14 homolog |
| evm.model.Scaffold500.59 | 3.77E-07 | 4 | *RFWD3* | E3 ubiquitin-protein ligase RFWD3 |
| evm.model.Scaffold63.167 | 8.16E-13 | 9 | *Klhdc4* | Kelch domain-containing protein 4 |
| evm.model.Scaffold221.37 | 3.28E-02 | 1 | *Lcmt2* | tRNA wybutosine-synthesizing protein 4 |
| evm.model.Scaffold212.78 | 7.66E-09 | 6 | *HAG3* | Elongator complex protein 3 |
| evm.model.Scaffold249.60 | 2.39E-05 | 3 | *LSMT-L* | [Fructose-bisphosphate aldolase]-lysine N-methyltransferase, chloroplastic |
| evm.model.Scaffold72.319 | 9.77E-05 | 3 | *Mettl16* | Methyltransferase-like protein 16 |
| evm.model.Scaffold25.194 | 2.22E-03 | 2 | *RIBA3* | Monofunctional riboflavin biosynthesis protein RIBA3, chloroplastic |
| evm.model.Scaffold117.193 | 1.42E-14 | 6 | *MCM4* | DNA replication licensing factor MCM4 |
| evm.model.Scaffold59.156 | 2.60E-11 | 6 | *RUS3* | Protein root UVB sensitive 3 |
| evm.model.Scaffold106.781 | 2.43E-04 | 1 | *Ycf3* | Ycf3-interacting protein 1, chloroplastic |
| evm.model.Scaffold182.100 | 1.59E-04 | 1 | *RRP6L3* | Protein RRP6-like 3 |
| evm.model.Scaffold283.473 | 3.62E-07 | 4 | *METTL13* | Methyltransferase-like protein 13 |
| evm.model.Scaffold170.200 | 4.60E-03 | 1 | NS | NS |
| evm.model.Scaffold57.166 | 2.95E-02 | 1 | *SURF1* | Surfeit locus protein 1 |
| evm.model.Scaffold112.5 | 2.61E-04 | 1 | *aroB* | 3-dehydroquinate synthase |
| evm.model.Scaffold321.97 | 3.66E-03 | 1 | *DJ1C* | Protein DJ-1 homolog C |
| evm.model.Scaffold140.159 | 0 | 21 | *CTN* | Cactin |
| evm.model.Scaffold397.12 | 3.90E-02 | 1 | NS | NS |
| evm.model.Scaffold167.467 | 1.73E-05 | 4 | *Ampp* | Probable Xaa-Pro aminopeptidase P |
| evm.model.Scaffold202.25 | 1.51E-06 | 4 | NS | NS |
| evm.model.Scaffold103.259 | 2.05E-08 | 3 | *OTP51* | Pentatricopeptide repeat-containing protein At2g15820, chloroplastic |
| evm.model.Scaffold64.783 | 9.28E-04 | 1 | *MSH2* | DNA mismatch repair protein MSH2 |

| **Table S16. Versions and main parameters of the software used in this study** | | |
| --- | --- | --- |
| Software | Version | Parameter |
| FALCON | 0.3.0 | length_cutoff, 11Kb; length_cutoff_pr, 11.5 Kb |
| PBJelly | PBSuite_14.9.9 | default |
| BWA | 0.7.12-r1039 | default |
| Pilon | Version 1.20 | --changes --vcf --diploid --fix bases --threads 32 --mindepth 10 |
| HISAT | 2.0.0-beta | hisat2 -N 1 |
| RepeatMasker | open-4.0.6 | RepeatMasker nolow -q -no_is -gff -norna -parallel 1 -engine wublast -species Viridiplantae |
| RepeatModeler | 1.0.4 | RepeatModeler -pa 30 -database zhongrenkeyuan.contigs.2.fasta -engine wublast |
| tRNAscan-SE | 1.3.1 | tRNAscan-SE -i -o -m |
| RNAmmer | 1.2 | rnammer -S euk -m lsu,ssu,tsu -gff |
| GeneWise | wise2-4-1 | genewise -genesf -gff -sum |
| Augustus | v3.0 | augustus --UTR=off --gff3=on --genemodel=complete --strand=both --min_intron_len=15 |
| GeneID | v1.4 | geneid -3 -P arabidopsis.param.Aug_4_2004 |
| GlimmerHMM | 3.0.4 | default |
| SNAP | 2013/2/16 | default |
| MCMCTREE | PAML v4.9e | clock=2, RootAge≤1.73, model=7, BDparas =110, kappa_gamma = 62, alpha_gamma = 11, rgene_gamma = 23.18, sigma2_gamma = 14.5 |
| Codeml | PAML v4.9e | Model A: model = 2, NSsites = 2, fix_omega = 0；Model A1: model = 2, NSsites = 2, fix_omega = 1, omega = 1 |
| CAFE | v3.0 | load -p 0.05 -r 10000 ，lambda -s |
