# Supplementary material for: De novo genome assembly of the red silk cotton tree (Bombax ceiba)
Source: Gigascience. 2018 May 10;7(5):giy051. doi: 10.1093/gigascience/giy051 (PMC5967522; doi:10.1093/gigascience/giy051)
Supplement: Response_to_Reviewer_Comments_Original_Submission.pdf [file giy051_response_to_reviewer_comments_original_submission.pdf]

Author's Response To Reviewer Comments

Close

Dear editors and reviewers,
The manuscript "De novo genome assembly of the red silk cotton tree (Bombax ceiba)" (GIGA-D-18-00045) has been carefully revised. The major revisions are marked in red, and the language has been polished by "sees-editing Ltd". We appreciate the detailed, useful comments and suggestions from you and the reviewers.
As the editor suggested,
1. The paper is submitted as a Data Note, and as such we do not require in-depth biological analyses. However, I do agree with reviewer 2 (their point #4), that some information on important genes and pathways will help to demonstrate the usefulness of the dataset.
Answer: As the editor and the reviewer suggested, information on important genes and pathways will help to demonstrate the usefulness of our genome data. B. ceiba is an ecologically important plant which could survive in extreme climate conditions. We calculated the average Ka/Ks values and conducted a branch-site likelihood ratio test to identify positively selected genes which could contribute to the ecological adaptation of B. ceiba. Thirty-six genes were identified, and some annotated genes were reported to be associated with ecological adaption. Please see line 190 to 210 in the revised manuscript.
2. Regarding minor point # 3 of reviewer 2: " L177: It may not be good to say this. Please rewrite. " I am actually not quite sure myself what the reviewer refers to here, but if I understand correctly, they feel the wording in that line 177 comes across as a bit cumbersome; this is true, but I'd prefer to have as much detail on parameters etc in the methods section as possible, and you should definitely keep that useful information.
Answer: As the editor suggested, the information on parameters was very important. So we kept this information in the revised manuscript.
3. Another minor remark: In the introduction, you refer to the use in Traditional Chinese Medicine: "Moreover, studies ... have validated its traditional medicinal usage." As this is still an active area of research, I feel it would be more appropriate to write "studies.... have explored its traditional medicine usage" instead of "validated".
Answer: The suggestion has been taken seriously, and this sentence has been changed to "studies.... have explored its traditional medicine usage". Please check line 41.

The point by point answers to the comments and suggestions of the reviewers are below.
Reviewer: 1
1. For every software, please (if not in the manuscript, then in the supplementary text) provide the non default parameters that were used, if any.
Answer: The suggestion has been taken seriously, and the versions and parameters of software used in our study have been provided as one supplementary table. Please see Table S15 in the supplementary file.
2. Line 60 precise the type (SE, PE, MP) and number of cycle of the Illumina sequencing.
Answer: We accept the review's suggestion, and the sequencing strategy (PE) has been added. Please see line 60 in the revised manuscript.
3. Line 106 - I would like some more details about the Illumina based scaffolding. Given that it's a small insert size library (400bp), how helpful was it to scaffold the PacBio assembly. Or do you mean the RNA-Seq data was used to scaffold? If that's the case, what parameters were used and how were multiple mapping case handled?
Answer: The sequencing data from the Illumina DNA library (400bp) was aligned against the genome assembly. As the reviewer stated, the small insert size library had relatively limited effects on

scaffolding. The analysis was aimed at filling gaps and correcting some sequencing errors of the genome assembly. Sorry for the ambiguity. The sentence has been corrected. Please see line 106 to 108.
4. Line 111 - I would replace the title there to: Evaluation of the completeness of the genome assembly gene space. BUSCO only look at the gene space. For the completeness of the genome assembly, one would need different metrics, such as FRC (feature response curve) or any quantification of the reads that do not align to the final assembly.
Answer: We take the review's suggestion, and the title has been changed to "Evaluation of the completeness of the genome assembly gene space".
5. Line 226 - There's a typesetting error in "financially"
Answer: We accepted the review's suggestion, and this error has been corrected in the revised manuscript.

Discretionary comments
1. Line 114 - Have you analysed the reads that do not align to the genome, what are these?
Answer: Unfortunately, we did not analyze the RNA reads which could not align to the genome assembly, and we did not know the details on these reads.
2. Line 116 - Have you checked what the 5.6% of incomplete BUSCO genes are?
Answer: There are 63 missing BUSCO genes and 19 incomplete BUSCO genes in total. We have searched these genes against the OrthoDB database to infer the gene functions.
For the 63 missing genes, there are eight genes falling in the category of pentatricopeptide repeat (PPR) superfamily protein, seven genes belonging to protein kinase domain, six genes annotated as transmembrane: Helical, five genes belonging to the tetratricopeptide repeat (TPR)-like superfamily proteins, and five genes annotated with unknown functions. The rest of genes were annotated with a variety of functions, such as peptidyl-prolyl cis-trans isomerase, WD40 repeat, regulator of chromosome condensation (RCC1) family protein, and so on.
For the 19 incomplete genes, two genes were characterized as transmembrane: Helical, and two genes were annotated with unknown functions. The other genes were annotated with a variety of functions, such as Zinc finger (C2H2), Reticulon-like protein, and so on.
3. Line 152 - The annotation could be refined using PASA. This would add splice variants to the annotation. Also, the PASA output can be post-processed to identify lncRNA. Many tools now allow for an in-depth analysis of differential transcript expression, differential transcript usage, etc. leveraging from more complete annotations.
Answer: As the reviewer suggested, PASA was a useful tool, especially for annotating splice variants. And we will try this software in our future study.

Reviewer: 2
1. Authors used a Kmer-based method to estimate the genome size. However, it seems the estimated genome size is smaller than their assembly size. Do authors know what happens here?
Answer: As the reviewer pointed, there was some inconstancy between the size of the genome survey and the genome assembly. As the heterozygosity rate of the B. ceiba genome was 0.88%, heterozygous regions which could not be assembled into consensus sequences might result in larger assembly. Besides, the Kmer-based method was just a preliminary estimation of the genome size. There could be some small deviation from the genome assembly.
2. For contamination checking, do authors believe a visualised GC content plot and the average GC content can tell there is no contamination in the assembly? Why didn't authors use other methods to check, such as BLASTN?
Answer: There were two strategies for deducing contaminations by GC content. 1. There were uneven GC contents in the genome. 2. The GC content was constant, but the sequencing depth varied among sequences. BLASTN was also a useful method for assessing contaminations. The suggestion of the reviewer has been taken seriously, and we randomly selected some sequences

from the genome assembly and searched the data against the NCBI database of bacteria with BLASTN. No contamination from bacteria was detected. Please check line 88 to 91 in the manuscript.
3. From the report, there is a ~150Mb difference between the BioNano consensus maps and the PacBio assembly. Does that mean there are repeat collapses in the PacBio assembly or some genome regions cannot be covered by PacBio reads? How does it look like in the comparison between BioNano consensus maps and the PacBio assembly? Do they align well?
Answer: The BioNano consensus genome map recorded the order of sequence fragments and the gap size between adjacent contigs. The BioNano genome map included gaps (Ns), whereas contigs of the primary PacBio assembly did not contain any gaps. It might be the reason why there was a ~150Mb difference. And the inconstancy between BioNano genome map and genome assembly size was also observed in other species, such as Fragaria vesca [1] and Tribolium castaneum [2]. With the initial PacBio genome assembly as reference, approximately 54 Gb out of 160 Gb BioNano clean data could be assigned to the genome map, and the effective coverage of assembly was about 60 X.

Reference
1. Edger PP, VanBuren R, Colle M, et al. Single-Molecule Sequencing and Optical Mapping Yields an Improved Genome of Woodland Strawberry (Fragaria Vesca) with Chromosome-Scale Contiguity. GigaScience. 2018;7 (2):1-7. doi:10.1093/gigascience/gix124.
2. Shelton JM, Coleman MC, Herndon N, et al. Tools and Pipelines for Bionano Data: Molecule Assembly Pipeline and Fasta Super Scaffolding Tool. BMC Genomics. 2015;16 (1):734.

4. This research is more likely a technical and bioinformatics report. Some biological stories, such as important genes, their functions and roles in certain pathways, are missing. How can this research help to improve Bombax ceiba's economic and ecological values?
Answer: As the reviewer suggested, information on important genes and pathways will help to demonstrate the usefulness of our genome data. B. ceiba is an ecologically important plant which could survive in extreme climate conditions. So we calculated the average Ka/Ks values and conducted a branch-site likelihood ratio test to identify positively selected genes which could contribute to the ecological adaptation of B. ceiba. Thirty-six genes were identified, and some annotated genes were reported to be associated with ecological adaption. Please see line 190 to 210 in the revised manuscript.
5. The English in the manuscript needs a further edit
Answer: We accepted the reviewer's suggestion, and the language has been polished by "sees-editing Ltd".
Minor:
1. It would be good to specify the version of each tool and detail the parameter settings.
Answer: We accepted the reviewer's suggestion, and the versions and parameters of software used in our manuscript were provided as one supplementary table. Please see Table S15 in the supplementary file.
2. It would be good to map the Illumina short reads (DNA) back to the assembly and give a mapping rate to inform the quality of the assembly.
Answer: We accepted the reviewer's suggestion, and we have mapped the Illumina short reads (DNA) back to the assembly. A mapping rate of 99.2% was achieved Please check line 106 to 108 in the revised manuscript.
3. L177: It may not be good to say this. Please rewrite.
Answer: As the reviewer suggested, we rewrote part of the sentence. As the detail on parameters was very important for this analysis, we kept this information in the revised manuscript. Please check line 178 in the manuscript.
4. FigureS4 and TableS4 are not informative. May change them to a table to list the stats of the raw BioNano maps.

Answer: We took the reviewer’s suggestion, and FigureS4 was removed. TableS4 was changed to a table which included statistics of the raw BioNano maps.
5. TableS5 may add two more columns: one is for the PacBio assembly, and the other one is for the BioNano optical scaffolding. From the improved table, readers can easily tell the changes before and after BioNano scaffolding.
Answer: We took the reviewer’s suggestion, and this information was added to TableS5. Please check Table S5 in the supplementary file.

Finally, we appreciate much your time in editing our manuscript and the reviewers for their valuable suggestions and comments. We look forward to hear your final decision when it is made.

Close
