# Supplementary material for: De novo genome assembly of the red silk cotton tree (Bombax ceiba)
Source: Gigascience. 2018 May 10;7(5):giy051. doi: 10.1093/gigascience/giy051 (PMC5967522; doi:10.1093/gigascience/giy051)
Supplement: Response_to_Reviewer_Comments_Revision_1.pdf [file giy051_response_to_reviewer_comments_revision_1.pdf]

Author's Response To Reviewer Comments

Close

Dear editor and reviewers,
The manuscript "De novo genome assembly of the red silk cotton tree (Bombax ceiba)" (GIGA-D-18-00045R1) has been carefully revised according to the reviewers' suggestion. The major revisions are marked in red.

Reviewer #2:
1. For my previous question 1: the authors pointed out that heterozygosity may affect the estimation of genome size by using Kmers. Do authors believe <1% heterozygosity rate can lead to ~100 Mb assembly differences (the final assembly is 895Mb)? Is that possible 17-mer underestimated the genome size (I understand that in BGI's paper they used 17-mer to estimate giant panda's genome size. Is 17-mer suitable for B.ceiba? If authors test different kmers, I suppose you will have different results).
Answer: As the reviewer suggested, we reanalyzed the genome size with other K-mers (19-mer and 21-mer). The estimated genome size was 835 Mb (19-mer) and 821 Mb (21-mer), respectively. The results did not dramatically depart from the genome size estimated with 17-mer (809 Mb). And our previously study of flow cytometry also suggested that the genome size of B. ceiba was approximately 800Mb (2C =1.55±0.03pg) [1]. The heterozygosity rate of B. ceiba genome was 0.88%. As pointed by many researchers, genomes with heterozygosity rate higher than 0.5% are considered as highly heterozygous [2]. Assembling highly heterozygous diploid genomes is a substantial challenge, and heterozygous regions could not be assembled into consensus may result in larger assembly [2]. So we concluded that the highly heterozygous genome of B. ceiba might be the main reason why there were ~100 Mb differences between the estimated genome size and the final assembly.

2. For my previous question 2: I appreciate that authors used BLASTN to confirm contaminations. However, shouldn't authors use the non-plant database instead of bacteria? Why did authors randomly select some contigs (how many?) instead of all of them? I understand that using random selection to avoid bias, but since the contamination rate is low (I suppose), you will have less chance to select a contamination contig if you only select a few contigs from the pool.
Answer: We accepted the reviewer's suggestion, and we searched all sequences of the genome assembly against the NCBI nucleotide collection (Nt) with BLASTN with E-value < 1e-5 and sequence identity > 70%. In total, 2494 significant hits were achieved. The top-hit species were Theobroma cacao and Gossypium species, comprising more than 69% of the hits (1733 hits). Only five hits from four non-plant species (Psyllidae sp., Trioza eugeniae, Diptacus sp., and Dichorragia nesimachus) were detected, which suggested there was no potential contamination from non-plant species in the genome of Bombax ceiba.
Please see line 89-95 in the revised manuscript and Table S4 in the supplementary file.

3. For my previous question 3: I understand that there are some discrepancies between Bionano consensus maps and the NGS reference. I want to know how many Bionano consensus maps can align to the Pacbio assembly or the total size of the aligned PacBio assembly? Since Bionano gives 1.09Gb consensus maps, does that mean the real genome size of B.ceiba is around 1.09 Gb? Does that mean the Pacbio assembly is still underestimated? For the 'Ns' added through Bionano scaffolding, it can be easily checked.
Answer: Approximately 64.3 Gb (2023 individual maps) out of 160 Gb BioNano clean data could be mapped to the Pacbio assembly, and the average molecule coverage depth of the genome map was

27 × (Please check line 106-108 in the revised manuscript). The final genome assembly scaffolded by BioNano mapping contained 386 gaps (25,395,219 bp in total). The larger genome consensus map (1.09 Gb) should mainly be contributed to redundancies because of the high genome heterozygosity.

New questions:
1. why did authors change the final assembled genome size from 869Mb to 895Mb, but didn't change any stats, is anything wrong with the previous calculation?
Answer: We are sorry for this matter. During the initial submission, we made a mistake and took the contig size (869Mb) as the assembled genome size due to negligence. So we changed the size of the final genome assembly to 895Mb (the scaffold size) in the revised manuscript. We appreciate very much for this comment.
2. From the density of Bionano label, it seems it is low. Which enzyme did authors use to generate the Bionano data? Is that Nt. BspQI?

Answer: We used Nt. BspQI enzyme in the BioNano mapping procedure. This information has been added to the revised manuscript. Please see line 98-99.

Finally, we appreciate the quick, detailed, useful comments and suggestions from the reviewers for improving our manuscript.

Reference

1. Zhou ZL, Xiong Z, Huan-Cheng MA, et al. Genome Sizes of Two Bombacaceae Plants. Journal of West China Forestry Science. 2014;43 (2):97-101. (in Chinese)
2. Kajitani R, Toshimoto K, Noguchi H, et al. Efficient De Novo Assembly of Highly Heterozygous Genomes from Whole-Genome Shotgun Short Reads. Genome Research. 2014;24 (8):1384-95.

Close
