# Supplementary material for: De novo genome assembly of the red silk cotton tree (Bombax ceiba)
Source: Gigascience. 2018 May 10;7(5):giy051. doi: 10.1093/gigascience/giy051 (PMC5967522; doi:10.1093/gigascience/giy051)
Supplement: Reviewer_1_Report_(Original_Submission) -- Nicolas Delhomme, Ph. D. rer. nat. — 2/23/2018 Reviewed [file giy051_reviewer_1_report_(original_submission).pdf]

Reviewer Report

Title: **De novo genome assembly of the red silk cotton tree (Bombax ceiba)**

Version: **Original Submission** **Date:** 2/23/2018

Reviewer name: **Nicolas Delhomme, Ph. D. rer. nat.**

Reviewer Comments to Author:

In their manuscript, Gao Yong et al., report on the genome assembly of the red silk cotton tree. Their analysis comprehensive, well conducted and well presented. The only comments I have are of minor or discretionary nature. Minor commentsFor every software, please (if not in the manuscript, then in the supplementary text) provide the non default parameters that were used, if any.Line 60 precise the type (SE, PE, MP) and number of cycle of the Illumina sequencing.Line 106 - I would like some more details about the Illumina based scaffolding. Given that it's a small insert size library (400bp), how helpful was it to scaffold the PacBio assembly. Or do you mean the RNA-Seq data was used to scaffold? If that's the case, what parameters were used and how were multiple mapping case handled?Line 111 - I would replace the title there to: Evaluation of the completeness of the genome assembly gene space. BUSCO only look at the gene space. For the completeness of the genome assembly, one would need different metrics, such as FRC (feature response curve) or any quantification of the reads that do not align to the final assembly.Line 226 - There's a typesetting error in "financially"Discretionary commentsLine 114 - Have you analysed the reads that do not align to the genome, what are these?Line 116 - Have you checked what the 5.6% of incomplete BUSCO genes are?Line 152 - The annotation could be refined using PASA. This would add splice variants to the annotation. Also, the PASA output can be post-processed to identify lncRNA. Many tools now allow for an in-depth analysis of differential transcript expression, differential transcript usage, etc. leveraging from more complete annotations.This is a signed review by Dr. Nicolas Delhomme, researcher at the Swedish University for Agricultural Sciences, and manager of the bioinformatics facility at the Umeå Plant Science Centre.

Level of Interest

Please indicate how interesting you found the manuscript: An article whose findings are important to those with closely related research interests

Quality of Written English

Please indicate the quality of language in the manuscript: Acceptable

Declaration of Competing Interests

Please complete a declaration of competing interests, considering the following questions:

- Have you in the past five years received reimbursements, fees, funding, or salary from an organisation that may in any way gain or lose financially from the publication of this manuscript, either now or in the future?
- Do you hold any stocks or shares in an organisation that may in any way gain or lose financially from the publication of this manuscript, either now or in the future?

- Do you hold or are you currently applying for any patents relating to the content of the manuscript?
- Have you received reimbursements, fees, funding, or salary from an organization that holds or has applied for patents relating to the content of the manuscript?
- Do you have any other financial competing interests?
- Do you have any non-financial competing interests in relation to this paper?

If you can answer no to all of the above, write 'I declare that I have no competing interests' below. If your reply is yes to any, please give details below.

I declare that I have no competing interests

I agree to the open peer review policy of the journal. I understand that my name will be included on my report to the authors and, if the manuscript is accepted for publication, my named report including any attachments I upload will be posted on the website along with the authors' responses. I agree for my report to be made available under an Open Access Creative Commons CC-BY license (http://creativecommons.org/licenses/by/4.0/). I understand that any comments which I do not wish to be included in my named report can be included as confidential comments to the editors, which will not be published.

I agree to the open peer review policy of the journal

To further support our reviewers, we have joined with Publons, where you can gain additional credit to further highlight your hard work (see: https://publons.com/journal/530/gigascience). On publication of this paper, your review will be automatically added to Publons, you can then choose whether or not to claim your Publons credit. I understand this statement.

Yes
