# Supplementary material for: De novo genome assembly of the red silk cotton tree (Bombax ceiba)
Source: Gigascience. 2018 May 10;7(5):giy051. doi: 10.1093/gigascience/giy051 (PMC5967522; doi:10.1093/gigascience/giy051)
Supplement: Reviewer_2_Report_(Original_Submission) -- Yuxuan Yuan — 2/27/2018 Reviewed [file giy051_reviewer_2_report_(original_submission).pdf]

Reviewer Report

Title: **De novo genome assembly of the red silk cotton tree (Bombax ceiba)**

Version: **Original Submission** **Date:** 2/27/2018

Reviewer name: **Yuxuan Yuan**

Reviewer Comments to Author:

Gao and colleagues present a draft Bombax ceiba genome assembly using PacBio sequencing, BioNano optical mapping and Illumina sequencing. From genome assembly, annotation and gene analyses, different methods were applied to scrutinise the quality of the assembly. However, before the acceptance of this manuscript I have some concerns and suggestions:Major: 1. Authors used a Kmer-based method to estimate the genome size. However, it seems the estimated genome size is smaller than their assembly size. Do authors know what happens here? 2. For contamination checking, do authors believe a visualised GC content plot and the average GC content can tell there is no contamination in the assembly? Why didn't authors use other methods to check, such as BLASTN?3. From the report, there is a ~150Mb difference between the BioNano consensus maps and the PacBio assembly. Does that mean there are repeat collapses in the PacBio assembly or some genome regions cannot be covered by PacBio reads? How does it look like in the comparison between BioNano consensus maps and the PacBio assembly? Do they align well? 4. This research is more likely a technical and bioinformatics report. Some biological stories, such as important genes, their functions and roles in certain pathways, are missing. How can this research help to improve Bombax ceiba's economic and ecological values?5. The English in the manuscript needs a further editMinor: 1. It would be good to specify the version of each tool and detail the parameter settings.2. It would be good to map the Illumina short reads (DNA) back to the assembly and give a mapping rate to inform the quality of the assembly.3. L177: It may not be good to say this. Please rewrite. 4. FigureS4 and TableS4 are not informative. May change them to a table to list the stats of the raw BioNano maps.5. TableS5 may add two more columns: one is for the PacBio assembly, and the other one is for the BioNano optical scaffolding. From the improved table, readers can easily tell the changes before and after BioNano scaffolding.

Level of Interest

Please indicate how interesting you found the manuscript: An article whose findings are important to those with closely related research interests

Quality of Written English

Please indicate the quality of language in the manuscript: Needs some language corrections before being published

Declaration of Competing Interests

Please complete a declaration of competing interests, considering the following questions:

- Have you in the past five years received reimbursements, fees, funding, or salary from an organisation that may in any way gain or lose financially from the publication of this manuscript, either now or in the future?

- Do you hold any stocks or shares in an organisation that may in any way gain or lose financially from the publication of this manuscript, either now or in the future?
- Do you hold or are you currently applying for any patents relating to the content of the manuscript?
- Have you received reimbursements, fees, funding, or salary from an organization that holds or has applied for patents relating to the content of the manuscript?
- Do you have any other financial competing interests?
- Do you have any non-financial competing interests in relation to this paper?

If you can answer no to all of the above, write 'I declare that I have no competing interests' below. If your reply is yes to any, please give details below.

I declare that I have no competing interests

I agree to the open peer review policy of the journal. I understand that my name will be included on my report to the authors and, if the manuscript is accepted for publication, my named report including any attachments I upload will be posted on the website along with the authors' responses. I agree for my report to be made available under an Open Access Creative Commons CC-BY license (http://creativecommons.org/licenses/by/4.0/). I understand that any comments which I do not wish to be included in my named report can be included as confidential comments to the editors, which will not be published.

I agree to the open peer review policy of the journal

To further support our reviewers, we have joined with Publons, where you can gain additional credit to further highlight your hard work (see: https://publons.com/journal/530/gigascience). On publication of this paper, your review will be automatically added to Publons, you can then choose whether or not to claim your Publons credit. I understand this statement.

Yes
