# Supplementary material for: De novo genome assembly of the red silk cotton tree (Bombax ceiba)
Source: Gigascience. 2018 May 10;7(5):giy051. doi: 10.1093/gigascience/giy051 (PMC5967522; doi:10.1093/gigascience/giy051)
Supplement: Reviewer_2_Report_(Revision_1) -- Yuxuan Yuan — 3/19/2018 Reviewed [file giy051_reviewer_2_report_(revision_1).pdf]

Reviewer Report

Title: **De novo genome assembly of the red silk cotton tree (Bombax ceiba)**

Version: **Revision 1** **Date:** 3/19/2018

Reviewer name: **Yuxuan Yuan**

Reviewer Comments to Author:

Thank authors for improving the manuscript and providing a quick response to my comments. Based on the reply I still have some concerns and suggestions. For my previous question 1: the authors pointed out that heterozygosity may affect the estimation of genome size by using Kmers. Do authors believe <1% heterozygosity rate can lead to ~100 Mb assembly differences (the final assembly is 895Mb)? Is that possible 17-mer underestimated the genome size (I understand that in BGI's paper they used 17-mer to estimate giant panda's genome size. Is 17-mer suitable for B.ceiba? If authors test different kmers, I suppose you will have different results). For my previous question 2: I appreciate that authors used BLASTN to confirm contaminations. However, shouldn't authors use the non-plant database instead of bacteria? Why did authors randomly select some contigs (how many?) instead of all of them? I understand that using random selection to avoid bias, but since the contamination rate is low (I suppose), you will have less chance to select a contamination contig if you only select a few contigs from the pool.For my previous question 3: I understand that there are some discrepancies between Bionano consensus maps and the NGS reference. I want to know how many Bionano consensus maps can align to the Pacbio assembly or the total size of the aligned PacBio assembly? Since Bionano gives 1.09Gb consensus maps, does that mean the real genome size of B.ceiba is around 1.09 Gb? Does that mean the Pacbio assembly is still underestimated? For the 'Ns' added through Bionano scaffolding, it can be easily checked.New questions: 1. why did authors change the final assembled genome size from 869Mb to 895Mb, but didn't change any stats, is anything wrong with the previous calculation?2. From the density of Bionano label, it seems it is low. Which enzyme did authors use to generate the Bionano data? Is that Nt. BspQI?

Level of Interest

Please indicate how interesting you found the manuscript: An article whose findings are important to those with closely related research interests

Quality of Written English

Please indicate the quality of language in the manuscript: Acceptable

Declaration of Competing Interests

Please complete a declaration of competing interests, considering the following questions:

- Have you in the past five years received reimbursements, fees, funding, or salary from an organisation that may in any way gain or lose financially from the publication of this manuscript, either now or in the future?
- Do you hold any stocks or shares in an organisation that may in any way gain or lose financially from the publication of this manuscript, either now or in the future?

- Do you hold or are you currently applying for any patents relating to the content of the manuscript?
- Have you received reimbursements, fees, funding, or salary from an organization that holds or has applied for patents relating to the content of the manuscript?
- Do you have any other financial competing interests?
- Do you have any non-financial competing interests in relation to this paper?

If you can answer no to all of the above, write 'I declare that I have no competing interests' below. If your reply is yes to any, please give details below.

I declare that I have no competing interests

I agree to the open peer review policy of the journal. I understand that my name will be included on my report to the authors and, if the manuscript is accepted for publication, my named report including any attachments I upload will be posted on the website along with the authors' responses. I agree for my report to be made available under an Open Access Creative Commons CC-BY license (http://creativecommons.org/licenses/by/4.0/). I understand that any comments which I do not wish to be included in my named report can be included as confidential comments to the editors, which will not be published.

I agree to the open peer review policy of the journal

To further support our reviewers, we have joined with Publons, where you can gain additional credit to further highlight your hard work (see: https://publons.com/journal/530/gigascience). On publication of this paper, your review will be automatically added to Publons, you can then choose whether or not to claim your Publons credit. I understand this statement.

Yes
